# The landscape of rare genetic variation associated with inflammatory bowel disease and Parkinson’s disease comorbidity

**DOI:** 10.1186/s13073-024-01335-2

**Published:** 2024-05-14

**Authors:** Meltem Ece Kars, Yiming Wu, Peter D. Stenson, David N. Cooper, Johan Burisch, Inga Peter, Yuval Itan

**Affiliations:** 1 The Charles Bronfman Institute for Personalized Medicine, Icahn School of Medicine at Mount Sinai, New York, NY 10029 USA; 2 College of Life Science, China West Normal University, Nan Chong, Si Chuan 637009 China; 3 Institute of Medical Genetics, Cardiff University, Cardiff, CF14 4XN UK; 4 Gastrounit, Medical Division, Copenhagen University Hospital – Amager and Hvidovre, Kettegård Alle 30, Hvidovre, Copenhagen, 2650 Denmark; 5 Copenhagen Center for Inflammatory Bowel Disease in Children, Adolescents and Adults, Copenhagen University Hospital – Amager and Hvidovre, Kettegård Alle 30, Hvidovre, Copenhagen, 2650 Denmark; 6 Department of Clinical Medicine, Faculty of Health and Medical Sciences, University of Copenhagen, Blegdamsvej 3B, Copenhagen, 2200 Denmark; 7 Department of Genetics and Genomic Sciences, Icahn School of Medicine at Mount Sinai, New York, NY 10029 USA; 8 Mindich Child Health and Development Institute, Icahn School of Medicine at Mount Sinai, New York, NY 10029 USA

**Keywords:** Crohn’s disease, Genetic pleiotropy, Inflammatory bowel disease, Parkinson’s disease, Ulcerative colitis, Whole genome sequencing

## Abstract

**Background:**

Inflammatory bowel disease (IBD) and Parkinson’s disease (PD) are chronic disorders that have been suggested to share common pathophysiological processes. *LRRK2* has been implicated as playing a role in both diseases. Exploring the genetic basis of the IBD-PD comorbidity through studying high-impact rare genetic variants can facilitate the identification of the novel shared genetic factors underlying this comorbidity.

**Methods:**

We analyzed whole exomes from the Bio*Me* BioBank and UK Biobank, and whole genomes from a cohort of 67 European patients diagnosed with both IBD and PD to examine the effects of *LRRK2* missense variants on IBD, PD and their co-occurrence (IBD-PD). We performed optimized sequence kernel association test (SKAT-O) and network-based heterogeneity clustering (NHC) analyses using high-impact rare variants in the IBD-PD cohort to identify novel candidate genes, which we further prioritized by biological relatedness approaches. We conducted phenome-wide association studies (PheWAS) employing Bio*Me* BioBank and UK Biobank whole exomes to estimate the genetic relevance of the 14 prioritized genes to IBD-PD.

**Results:**

The analysis of *LRRK2* missense variants revealed significant associations of the G2019S and N2081D variants with IBD-PD in addition to several other variants as potential contributors to increased or decreased IBD-PD risk. SKAT-O identified two significant genes, *LRRK2* and *IL10RA*, and NHC identified 6 significant gene clusters that are biologically relevant to IBD-PD. We observed prominent overlaps between the enriched pathways in the known IBD, PD, and candidate IBD-PD gene sets. Additionally, we detected significantly enriched pathways unique to the IBD-PD, including MAPK signaling, LPS/IL-1 mediated inhibition of RXR function, and NAD signaling. Fourteen final candidate IBD-PD genes were prioritized by biological relatedness methods. The biological importance scores estimated by protein–protein interaction networks and pathway and ontology enrichment analyses indicated the involvement of genes related to immunity, inflammation, and autophagy in IBD-PD. Additionally, PheWAS provided support for the associations of candidate genes with IBD and PD.

**Conclusions:**

Our study confirms and uncovers new *LRRK2* associations in IBD-PD. The identification of novel inflammation and autophagy-related genes supports and expands previous findings related to IBD-PD pathogenesis, and underscores the significance of therapeutic interventions for reducing systemic inflammation.

**Supplementary Information:**

The online version contains supplementary material available at 10.1186/s13073-024-01335-2.

## Background

Inflammatory bowel disease (IBD) comprises a group of chronic inflammatory diseases that primarily affect the gastrointestinal tract, including Crohn’s disease (CD) and ulcerative colitis (UC). CD is distinguished by segmental transmural inflammation affecting the ileum and colon, whereas UC is usually limited to the colon, and characterized by mucosal inflammation [1]. Parkinson’s disease (PD) is one of the most common neurodegenerative disorders, presenting with bradykinesia, rigidity, resting tremor, and postural instability [2]. It is characterized by the progressive loss of dopaminergic neurons in the substantia nigra and the presence of intracellular protein aggregates known as Lewy bodies [3]. Emerging evidence suggests a link between these two apparently unrelated disorders, indicating shared risk factors and underlying pathophysiology that is consistent with the “gut-brain axis” hypothesis [4–8]. IBD-PD co-occurrence has been attributed to neurodegeneration driven by chronic intestinal inflammation and pleiotropic genetic factors [4]. A recent meta-analysis, involving 12 million patients from 9 observational studies, provided further support for previous findings, indicating that both CD and UC are associated with an increased risk of PD diagnosis [9]. This increased risk was particularly prominent in older patients (> 65 years old), irrespective of gender. Moreover, exposure to anti-inflammatory tumor necrosis factor-α inhibitors in IBD patients has been linked to a significant reduction in PD risk [6, 9]. These findings suggest the involvement of inflammation-mediated processes and/or potentially shared genetic factors underlying both IBD and PD.

The most well-established gene implicated in the IBD-PD pleiotropy is leucine-rich repeat kinase 2 (*LRRK2*). Polymorphisms in *LRRK2* have been shown to be associated with both PD and CD, suggesting the impact of impaired autophagy in the pathogenesis of both conditions [5]. Variants that result in increased activity of LRRK2 have been shown to be associated with an elevated risk of developing both PD and CD, whilst a haplotype with a deactivating *LRRK2* mutation, R1398H, has been found to be associated with protection against CD [5] and PD [10–12]. However, despite genetic pleiotropy for some of the *LRRK2* variants (i.e., G2019S, N2081D, N551K, and R1398H) [5], each of these conditions is associated with specific *LRRK2* variants. For example, G2019S is the major genetic risk for PD [13], whereas N2081D is considered a risk for CD [5, 14]. Moreover, other strong genetic predictors of PD, such as R1441G/C/H, Y1699C, R1628P, G2385R, and I2020T, have been shown to be associated exclusively with PD [15], whereas M2397T was not linked to PD [16]. Therefore, it is not immediately clear whether any of these or other *LRRK2* variants may lead to IBD-PD comorbidity. Other than *LRRK2*, several other pleiotropic loci, including *MAPT*, *HLA*, *MHC*, *ATP6V0A1*, and *NOD2*, have been identified to be associated with PD, CD, UC, and other autoimmune disorders [4, 7, 8]. Previous studies that have examined the genetic pleiotropy between IBD and PD have primarily estimated the genetic correlation between these two conditions by means of genome-wide association study (GWAS) data from separate analyses of IBD and PD [7, 8]. However, conducting a joint analysis of individuals affected by both IBD and PD would provide important insights into the underlying mechanisms shared by these two conditions.

**Fig. 1:**
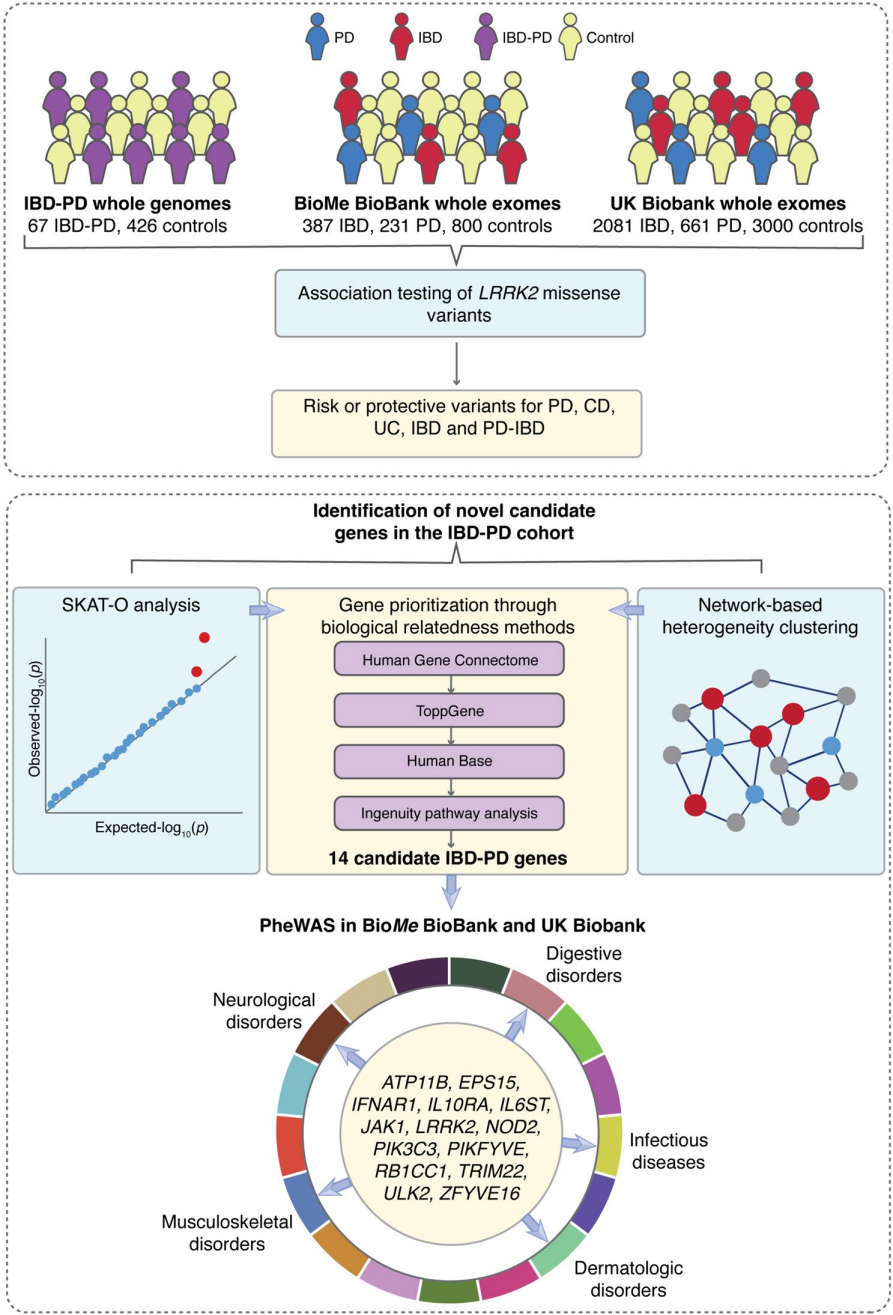
Study workflow. Parkinson’s disease (PD) and inflammatory bowel disease (IBD) cases were identified based on ICD-10 codes in the UK Biobank and Bio*Me* BioBank. The IBD-PD cohort comprised 67 individuals diagnosed with both PD and IBD and 426 healthy controls from the 1000 Genomes Project (1KGP). Variant-level association testing was performed separately in each cohort using *LRRK2* variants observed in the whole exome (UK Biobank and Bio*Me* BioBank) and whole genome sequencing (IBD-PD cohort) data (upper panel). Discovery of novel candidate genes was performed using the sequence kernel association test (SKAT-O) and network-based heterogeneity clustering (NHC) methods; the genes were further prioritized using pathway enrichment and biological relatedness methods. Fourteen prioritized candidate genes were then investigated in the Bio*Me* BioBank and UK Biobank using phenome-wide association analysis (PheWAS) to validate identified associations (lower panel)

Here, we investigated the effect of *LRRK2* missense variants on PD only, CD only, UC only, IBD only, and the co-occurrence of IBD and PD risk (IBD-PD) using sequencing data from the Danish Registry, the Mount Sinai Bio*Me* BioBank, and the UK Biobank. Furthermore, we performed a series of gene-level association and network-based analyses using high-impact rare variants in the IBD-PD cohort and prioritized candidate physiologically-relevant genes associated with this comorbidity. Finally, we conducted phenome-wide association studies (PheWAS) in Bio*Me* BioBank and UK Biobank to evaluate the pleiotropic effects of the candidate IBD-PD genes, as well as IBD-PD comorbidities (Fig. 1).

## Methods

### Study cohorts

#### IBD-PD cohort

We identified 76 patients diagnosed with both IBD and PD in the Danish National Biobank resource (https://www.danishnationalbiobank.com/) using the International Classification of Diseases (ICD) codes from the Danish National Patient Registry (Additional file 1: Identification of the IBD-PD samples in the Danish National Biobank). Owing to the regulations of the Danish Data Protection Agency, we were precluded from gathering demographic and clinical data regarding patients’ specific IBD subtypes or their ages at the time of biosample collection. Whole-genome sequencing (WGS) was performed using the Illumina HiSeq X platform with 2 × 150 bp paired-end reads. Alignment of the raw reads and variant calling were performed following the best practice guidelines of the Genome Analysis Toolkit (GATK) [17] (Additional file 1: Whole genome sequencing of the IBD-PD samples).

For the control group, high-coverage WGS data of 1000 Genomes Project Phase 3 (1KGP) individuals were downloaded on March 20, 2023, from the following URL: http://ftp.1000genomes.ebi.ac.uk/vol1/ftp/data_collections/1000G_2504_high_coverage/working/20190425_NYGC_GATK/raw_calls_updated/ [18]. This dataset comprised 427 samples from non-Finnish European populations. Joint genotyping and quality control (QC) steps were performed on this combined case–control dataset (Additional file 1: Genotyping and quality control of the IBD-PD cohort and Additional file 2: Table S1). After QC filtration, which led to the exclusion of 9 IBD-PD cases and 1 control, the final dataset comprised a total of 20,158,023 variants across 67 IBD-PD cases and 426 controls. Of these, 40 (59.7%) IBD-PD cases and 214 (50.2%) controls were males. Genetic ancestries of the IBD-PD cases were determined using principal component analysis (PCA) and ADMIXTURE [19] by utilizing 1KGP populations as a reference, which confirmed their European ancestry (Additional file 1: Determining the genetic ancestries of the IBD-PD cases, Fig. S1 and S2).

#### Bio*Me* BioBank

The Mount Sinai Bio*Me* BioBank comprises whole exome sequencing (WES) data from 31,250 participants who were recruited from Mount Sinai primary care clinics (https://icahn.mssm.edu/research/ipm/programs/biome-biobank). No specific criteria were used during the recruitment; therefore, the Bio*Me* participants should be a representative sample of New York City and its neighborhood. The genetic data of the participants are linked to their electronic health records (EHR), which we used to select IBD, CD, UC, and PD cases and controls. WES was performed by Regeneron (NY, USA) using the IDT xGen capture kit on an Illumina v4 HiSeq 2500 platform (a total of 8,761,478 sites across 31,250 samples). A QC check was performed to remove contaminated, low coverage, gender discordant, genotype-exome discordant, and duplicate samples. Variant sites with a missingness rate > 0.02, or allelic balance < 0.30 or > 0.80 were excluded from the dataset. The final dataset included 30,813 samples and 8,890,425 variants.

#### UK Biobank

The UK Biobank dataset is a comprehensive collection of health-related information gathered from over 500,000 participants across the United Kingdom [20]. WES data of 200,643 UK Biobank participants were used in the current study. A QC check was performed to select samples and variants of high quality, using the following steps using PLINK v.1.9 [21]. Samples with a missingness rate exceeding 5%, displaying gender discrepancies and duplicated samples were excluded. Second-degree or closer relatives were determined using KING and excluded to retain unrelated samples in the dataset [22]. Variants were removed if their missingness rate exceeded 20% or if they demonstrated a significant deviation from Hardy–Weinberg equilibrium (HWE) with a *P* < 1 × 10^−6^. Consequently, the final dataset comprised 189,448 samples 17,402,345 variants.

In both Bio*M*e BioBank and UK Biobank, individuals of European descent were genetically determined using HapMap3 populations [23] as the reference through an analysis with fastSTRUCTURE [24]. CD, UC, IBD, and PD cases and controls were identified based on ICD-9 and ICD-10 diagnoses of Bio*Me* BioBank and UK Biobank participants (Additional file 1: Identification of IBD and PD samples with European descent from Bio*Me* and UK Biobank). The final counts of PD, IBD, CD, and UC cases, as well as controls, are detailed in Table 1.

**Table 1:** Cohorts used in the variant-level analysis of *LRRK2*

Cohort	Phenotype	Case#	Control#	Sex, cases (F/M)	Sex, controls (F/M)
**IBD-PD**	IBD-PD	67	426	27/40	214/212
**Bio*Me* BioBank**	PD	231	800	93/138	441/359
	IBD	387	800	205/182	
	CD	209	800	116/93	
	UC	141	800	67/74	
**UK Biobank**	PD	661	3000	270/391	1734/1266
	IBD	2081	3000	1069/1012	
	CD	603	3000	350/253	
	UC	1282	3000	613/669	

### Variant annotation and filtration

To assess predicted impacts on gene products and population frequencies of the variants identified in the IBD-PD cohort, as well as in Bio*Me* Biobank and UK Biobank, variants were annotated using Variant Effect Predictor (VEP) v.106, CADD scores (v.1.6), and allele frequencies in gnomAD v2 and 1KGP populations [18, 25–27]. To control the false-negative rate of predicted deleterious mutations by CADD, a Mutation Significance Cutoff (MSC, https://lab.rockefeller.edu/casanova/MSC) was applied for each gene [28]. Additionally, genes were annotated with the Gene Damage Index (GDI, https://lab.rockefeller.edu/casanova/GDI), which serves as an indicator of genes that exhibit high polymorphism in the general healthy population and hence are less likely to be disease-associated [29].

For the identification of novel candidate genes associated with IBD-PD, variants were filtered based on their consequences as obtained from VEP annotation. High-impact variants (“start lost,” “stop lost,” “stop gained,” “splice_acceptor_variant,” “splice_donor_variant,” “protein_altering_variant,” “start_retained_variant,” “stop_retained_variant,” and ‘frameshift_variant”) and moderate-impact variants (“missense variants,” “inframe_insertion,” “inframe_deletion”) were extracted. Moderate-impact variants were further filtered if their CADD scores were greater than the lower boundary of the 95% confidence interval of the corresponding gene’s MSC. Further, variants in genes with a GDI value of less than 13.84 (the cutoff proposed for human diseases under the generalized model) were included [29]. Finally, variants with a maximum minor allele frequency (MAF) below 1% in gnomAD and 1KGP subpopulations were retained to identify the final set of presumably deleterious variants for further analyses. The same filtering steps according to MAF and GDI were applied to obtain synonymous variants for the neutral model, which estimates the degree of inflation due to population substructure or possible technical artifacts.

### Analysis of *LRRK2* variants

To conduct an in-depth characterization of the role of *LRRK2* missense variants in IBD-PD comorbidity, as well as to assess their effect on CD, UC, IBD, and PD, we extracted all *LRRK2* missense variants from the IBD-PD, Bio*Me* BioBank, and UK Biobank cohorts. PCA was performed using PLINK v1.9 with linkage disequilibrium (LD)-pruned variants (*r*^2^ = 0.2) with a MAF greater than 5% and not exceeding HWE with a *P* < 1 × 10^−6^ [21]. The selection of the number of PCs to be included as covariates in the analyses was based on the scree test [30] (Additional file 1: Fig. S3). The first two genetic principal components (PCs), sex and age, were used as covariates in the Bio*Me* BioBank analyses while PC1, sex, and age were employed as covariates in the UK Biobank analyses. For the IBD-PD cohort, PC1 and sex were used as covariates. For association testing of *LRRK2* missense variants with a minor allele count (MAC) ≥ 3 and a case MAC ≥ 1, we used Firth’s logistic regression method implemented in PLINK v.2.0 [21, 31]. Pairwise LDs of *LRRK2* missense variants were calculated using PLINK v.1.9.

### Sequence Kernel Association Test analysis and network-based heterogeneity clustering of the IBD-PD cohort

To identify novel genes associated with the IBD-PD comorbidity, we used the optimized method of the Sequence Kernel Association Test (SKAT-O) to accommodate rare variants with potentially different directions of effect [32]. SKAT-O is an optimal combination of the burden test and SKAT, aiming to enhance statistical power. The burden test combines minor alleles at variants in a region, assuming they have the same direction of effect, and compares the difference in allele frequencies between cases and controls. On the other hand, SKAT employs a regression framework and a variance-component test, allowing it to account for variants with different directions of effect. SKAT-O dynamically operates as the burden test when it surpasses SKAT in power, and functions as SKAT when SKAT exhibits greater power than the burden test [32]. Initially, we employed SKAT-O using a set of synonymous variants of 72 IBD-PD cases and 426 controls (Fig. 1), incorporating PC1 and sex as covariates [33]. The analysis revealed genes with significantly inflated *P* values (Additional file 1: Fig. S4A). Similarly, SKAT-O using presumably deleterious variants also displayed genes with inflated *P* values (Additional file 1: Fig. S4B). Further investigation into the cause of this inflation resulted in the removal of 5 IBD-PD cases that exhibited an excess number of heterozygous variants in these genes. Subsequent analysis with 67 IBD-PD cases and 426 controls resolved the inflation issue (Additional file 1: Fig. S4C and Fig. S4D). Among the analyzed genes, only *EPHA4* exhibited a significant burden of synonymous variants (*P* = 3.26 × 10^−6^). Subsequently, SKAT-O was performed using the set of presumably deleterious variants in the 67 IBD-PD cases and 426 controls, while employing the same covariates. The Bonferroni correction method was used to adjust *P* values so as to allow for multiple testing.

Additionally, we used the network-based heterogeneity clustering (NHC) algorithm to cluster cases and genes that harbor presumably deleterious variants by performing a gene cluster-based burden test. NHC was specifically tailored for small-sized cohorts to increase statistical power by utilizing a background protein–protein interaction (PPI) network to identify gene clusters significantly enriched in cases compared to controls [34]. In addition, NHC performs several pathway and ontology enrichment analyses for the identified gene clusters. The same set of presumably deleterious variants and covariates was used in this analysis to identify and prioritize candidate gene clusters that displayed significant enrichment in IBD-PD cases compared to controls.

### Gene prioritization via pathway and gene enrichment analysis

To capture additional potential gene candidates associated with IBD-PD, we performed pathway and gene enrichment analyses, relaxing the *P* value threshold to < 0.01 based on the SKAT-O results, resulting in 84 genes (Fig. 1). Additionally, we selected genes in significant clusters identified by NHC (cluster *P* < 0.05), except for those enriched for previously-identified control-embedded pathways, leaving us with 38 genes [34]. The resulting gene set comprised 120 unique genes, which were considered potential candidates associated with IBD-PD. To examine the genes that have a close biological relationship to the known IBD and PD genes, we generated sets of known genes. The set of 157 known IBD genes was generated based on studies of IBD, CD, and UC [35, 36] following the methodology described in our previous study [37]. We included 142 genes from the merged signals of the genome-wide significant loci (*P* < 5 × 10^−8^) from Jostins et al. [36], as well as 15 genes from the 95% credible set of the fine-mapping results by Huang et al. [35]. For the set of known PD genes, we extracted genes associated with PD from the Human Gene Mutation Database (HGMD) Professional v. 2022.4 [38], which is the largest database currently available of high-quality and manually curated pathogenic variants. We specifically used the HGMD disease-causing mutations with the highest confidence only based on experimental evidence (DM), resulting in the identification of 103 known PD genes. We employed Ingenuity Pathway Analysis (IPA, QIAGEN Inc., version 01–21-03 https://www.qiagenbioinformatics.com/products/ingenuity-pathway-analysis) to compare significantly enriched pathways in the three gene sets.

Then, by utilizing the human gene connectome (HGC, https://lab.rockefeller.edu/casanova/HGC) [39], we calculated the average biological distance of 120 IBD-PD-associated genes (*D*_candidate_) to 157 known IBD genes and 103 known PD genes. We compared *D*_candidate_ with *D*_random,_ which was derived from randomly sampled gene sets of equivalent size (*n* = 120) to calculate empirical *P* values using 10,000 resampling iterations (Additional file 1: Methods used in biological relatedness, pathway, and gene enrichment analysis).

We then employed four complementary methods of pathway and biological relatedness approaches: ToppGene [40], IPA, HumanBase [41], and the Human Gene Connectome (HGC) [39] to obtain the final 14 IBD-PD associated candidate genes that were selected by all four methods (Additional file 1: Methods used in biological relatedness, pathway, and gene enrichment analysis). Additionally, DM variants in the final 14 IBD-PD-associated genes were explored in the HGMD.

#### The biological importance scores

We evaluated the biological importance of candidate genes by counting the number of IBD and PD genes in significantly enriched pathways, PPI networks, and ontologies with a *P* value < 0.01. We utilized several tools and databases to calculate the scores, including IPA core pathway analysis, InnateDB pathway analysis, InnateDB Gene Ontology (GO) analysis, and STRING interactome from NetworkAnalyst [42, 43]. We calculated the IBD-specific, PD-specific, and combined IBD-PD scores by summing the scaled scores from each data source.

### PheWAS

Gene-level PheWAS was conducted in both the Bio*Me* BioBank and UK Biobank to validate the phenotypes associated with the 14 candidate IBD-PD genes, as well as to evaluate their pleiotropic effects (Fig. 1). The Bio*Me* BioBank includes samples from diverse ancestral origins (Additional file 2: Table S2), whereas the UK Biobank is primarily composed of individuals of European ancestry. ICD-9 (*n* = 1056 and 3344 in Bio*Me* Biobank and UK Biobank, respectively) and ICD-10 diagnoses (*n* = 19,085 and 11,727 in Bio*Me* Biobank and UK Biobank, respectively) of Biobank participants were converted into 1856 clinically relevant phenotypes (phecodes) using Phecode Map v1.2 [44]. 1376 and 1415 phecodes with at least 20 cases were used in the Bio*Me* and UK Biobank analyses, respectively. We used a combination of PheWAS and SKAT-O binary robust methods, employing a case–control ratio not exceeding 1:99 for each disease and trait [45, 46]. Only variants identified within the IBD-PD cohort were included. PCA was conducted using the same parameters as those used in the IBD-PD cohort. Age, biological sex, and PC1-10 were used as covariates in the analysis. The Bonferroni-adjusted phenome-wide *P* value was determined as 3.63 × 10^−5^ (0.05/1376) and 3.53 × 10^−5^ (0.05/1415) for Bio*Me* and UK Biobank analyses, respectively.

## Results

### Analysis of *LRRK2* variants in the IBD-PD, Bio*Me*, and UK Biobank cohorts

**Fig. 2:**
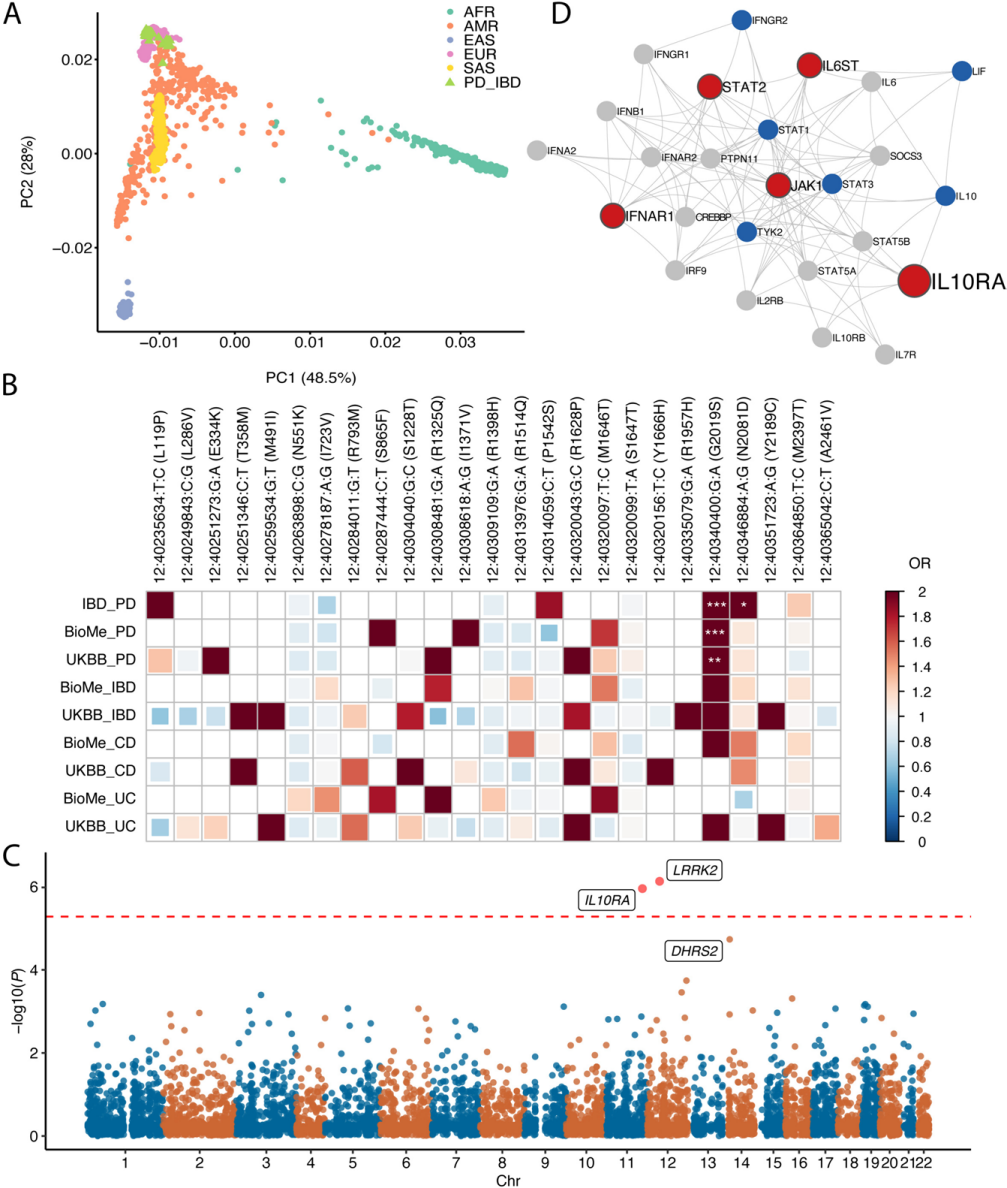
*LRRK2*-centered and genome-wide analysis of the IBD-PD comorbidity. **A** PCA plot demonstrating the distribution of IBD-PD cases among 1000 Genomes Project (1KGP) superpopulations. **B** Correlogram displaying the associations of *LRRK2* missense variants with Parkinson’s disease (PD), inflammatory bowel disease (IBD), Crohn’s disease (CD), and ulcerative colitis (UC) in the Danish Registry, Bio*Me* BioBank, and UK Biobank (UKBB) cohorts. The colors indicate odds ratios (ORs), whereas asterisks denote the significance level of the *P* values (* *P* < 0.05, ** *P* < 0.01, ***P < 0.001). Empty cells indicate that the variant had a minor allele count (MAC) < 3 in the respective cohort or MAC < 1 in cases, and hence the association could not be assessed. **C** Manhattan plot displaying the results of the SKAT-O analysis. The two genes that passed the level of genome-wide significance (*LRRK2* and *1L10RA*) are highlighted in red. The dashed line denotes the genome-wide significance level. **D** STRING protein–protein interaction (PPI) network generated using genes identified in the most significant cluster (cluster 7) in the NHC analysis (in red). The size of the nodes is proportional to the number of variant carriers. Known IBD-associated genes in the extended PPI network are highlighted in blue

The IBD-PD cohort comprised 67 individuals of Northwestern and Southern European ancestry diagnosed with both IBD and PD, along with 426 non-Finnish Europeans from the 1KGP serving as controls (Fig. 2A and Additional file 1: Fig. S1 and Fig. S2). We performed variant-level association tests to assess the impact of 9 *LRRK2* missense variants identified in the IBD-PD dataset on the IBD-PD phenotype. Additionally, we extracted a total of 14 *LRRK2* missense variants from the Bio*Me* cohort and 28 missense variants from the UK Biobank cohort and examined their associations with PD, IBD, CD, and UC (Fig. 2B, Additional file 2: Table S3 and Table S4). Our analysis identified the well-known PD and CD-associated variant, G2019S, as being significantly associated with the IBD-PD comorbidity (*P* = 1.59 × 10^−4^, Bonferroni-corrected *P* = 5.56 × 10^−3^) [5, 14, 47]. Moreover, G2019S showed an increased risk for PD in both the Bio*Me* and UK Biobank cohorts (*P* = 6.56 × 10^−5^ and 7.66 × 10^−3^, respectively). Additionally, the N2081D variant was associated with the IBD-PD comorbidity at a nominal significance level (*P* = 0.027).

We further investigated the odds ratios (ORs) derived from Firth’s regression for all 25 variants identified in the IBD-PD, Bio*Me*, and UK Biobank cohorts. The G2019S variant demonstrated an increase for PD and both IBD subtypes, whereas the N2081D and L119P variants were associated with an elevated risk for increased risk for PD and CD. In addition to G2019S, N2081D, and L119P, the P1542S and M2397T variants exhibited an increased risk for the combined IBD-PD phenotype. Interestingly, the P1542S variant was exclusively associated with an increased risk of IBD-PD comorbidity, whereas it exhibited a decreased risk of PD in the Bio*Me* BioBank analysis, despite displaying odds ratios close to 1 in the remaining analyses. Additionally, E334K, S865F, I1371V, R1325Q, R1628P, and M1646T variants displayed an increased risk for both PD and one or both subtype(s) of IBD based on the Bio*Me* and UK Biobank analyses. S1647T, previously considered as a neutral variant [48], displayed ORs close to 1 for both PD and IBD subtypes, as expected.

Afterwards, we searched these 25 *LRRK2* variants in HGMD and found that 16 variants had been reported to be associated with PD at varying confidence levels (Additional file 2: Table S5) [38]. Among those, 6 variants (E334K, R793M, S1228T, R1325Q, G2019S, and Y2189C) were associated with PD and classified under the DM category. Interestingly, the R793M, S1228T, and Y2189C variants demonstrated increased ORs for IBD but not for PD in the UK Biobank cohort, although the association of R793M and Y2189C with PD could not be assessed due to low MAC. The R1398H, N551K variants (the only variant pair in strong LD, *r*^2^ = 0.93) and I723V exhibited low ORs for the IBD-PD comorbidity. However, they displayed a positive relationship with UC and IBD risk in the Bio*Me* BioBank dataset. Overall, these results provide further evidence in support of the role of *LRRK2* variants in the pathogenesis of IBD-PD comorbidity. We note that while the reported OR results implicate trends of risk or protection, the majority of the *P* values in the aforementioned results (Fig. 2B and Additional file 2: Table S4) did not reach study-wide statistical significance, most likely due to the limited sample size of the study cohorts.

### Gene-level association testing using the IBD-PD cohort

To identify novel genes associated with the IBD-PD comorbidity, SKAT-O analyses were performed using 9561 genes containing 34,640 presumably deleterious rare variants (Fig. 2C, Additional file 2: Table S6). As a result, *LRRK2* (*P* = 7.41 × 10^−7^) and *IL10RA* (*P* = 1.11 × 10^−6^) attained the genome-wide significance level (Bonferroni-corrected *P* = 5.23 × 10^−6^). Consistent with its well-established association with both PD and IBD, *LRRK2* was identified as the most significant gene in the analyses [5]. Following *LRRK2* and *IL10RA*, the third most significant gene according to the SKAT-O results was *DHRS2* (*P* = 1.85 × 10^−5^). These findings confirmed that *LRRK2* is an IBD-PD-associated gene and revealed *IL10RA* (and potentially *DHRS2*) as candidate genes.

### Network-based heterogeneity clustering in the IBD-PD cohort

Genetic heterogeneity is a common feature in many human diseases, including IBD and PD [49, 50]. To overcome the challenges caused by genetic heterogeneity in identifying disease-associated genes through conventional frequency-based case–control studies that assume genetic homogeneity, we employed NHC to identify candidate gene clusters associated with IBD-PD [34]. NHC is optimized for case–control analyses of small cohorts by performing pathway-level aggregations of genes carrying candidate variants, employing a clustering approach of gene–gene biological relatedness metric. NHC revealed 12 gene clusters with a cluster *P* < 0.05 (Additional file 2: Table S7). Four significant gene clusters (clusters 68, 70, 71, and 76) were deprioritized because the significantly enriched pathways in these gene clusters had been previously found to be highly enriched in healthy European controls [34]. Two significant gene clusters (clusters 7 and 73) included genes previously associated with Parkinsonism (*FIG4* and *VAC14*) [51, 52] and IBD (*IFNAR1*, *IL6ST*, *ATG16L1*, and *NOD2*) [36] (Fig. 2D and Additional file 1: Fig. S5).

There were four additional potentially relevant significant gene clusters identified by NHC. Cluster 24 contained *ATP6V0A1*, *ATP6V0A2*, *ATP6V0D2*, *ATP6V1B1*, and *ATP6V1H*, genes encoding components of the vacuolar-ATPase complex, which plays a role in phagosome acidification. The vacuolar-ATPase complex has been implicated in the pathophysiology of several neurodegenerative diseases, including PD [53]. Additionally, previous GWAS have identified *ATP6V0A1* as a candidate for PD [54]. Cluster 20 consisted of genes involved in GMP biosynthesis, *GMPS*, *IMPDH1*, and *IMPDH2*. *IMPDH1* has been suggested to play a role in PD pathogenesis through protein misfolding and accumulation [55]. Furthermore, mutations in *IMPDH2* have been associated with a dominant-type juvenile-onset dystonia-tremor disorder [56]. Cluster 57 encompassed *EPS15*, *FCHO1*, *FCHO2*, and *HGS*, genes related to endocytosis. Deficiency of *FCHO1* has been shown to be associated with both IBD and Guillain Barré syndrome [57]. Additionally, *EPS15* encodes an endocytic accessory protein that interacts with parkin and might contribute to the pathogenesis of PD [58]. Lastly, cluster 40 contained *LIN9*, *MYBL2*, *RBL1*, and *TFDP1*. *TFDP1* has been found to be associated with IBD in a previous GWAS [59]. These findings highlight the potential relevance of these gene clusters to the pathogenesis of IBD and PD.

### Comparison of IBD-PD-associated genes with established IBD and PD genes

**Fig. 3:**
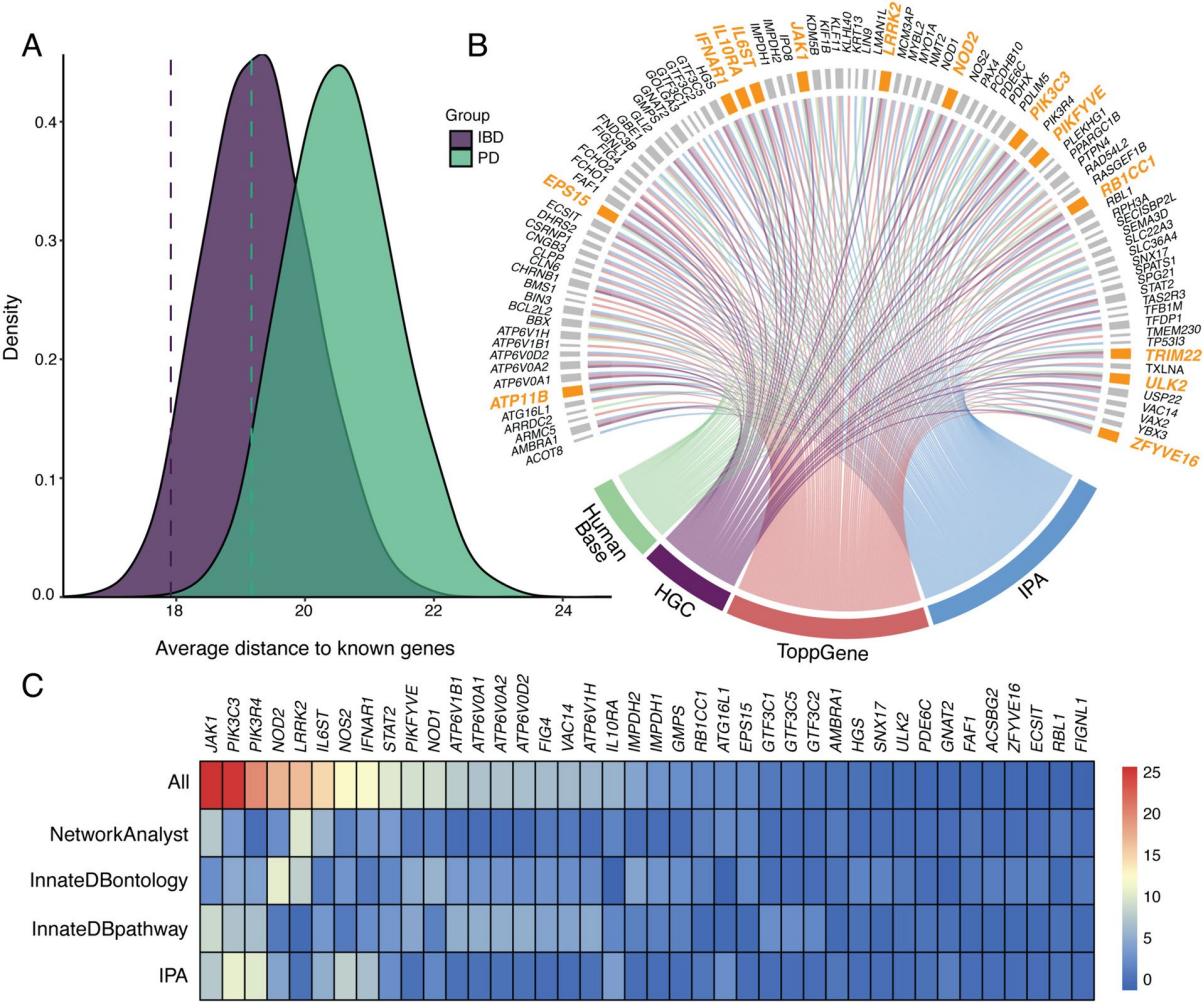
Candidate gene prioritization and validation using a phenotype-wide association study (PheWAS). **A** Density plots displaying the distribution of the average distances of 120 random genes (*D*_random_) to known inflammatory bowel disease (IBD, in purple) and known Parkinson’s disease (PD, in green) genes obtained from 10,000 resampling iterations. Dashed lines represent the average distance of the candidate gene set (*D*_candidate_, *n* = 120) to known IBD and PD genes. **B** Circos plot showing the candidate genes identified by four complementary pathway and biological relatedness approaches. Final candidate genes that were identified by all four methods are highlighted in orange. **C** Heatmap of the combined biological importance scores of the IBD-PD candidate genes. A higher score indicates a higher number of shared pathways, ontologies, or modules with known IBD and PD genes. The colors represent the magnitude of the scaled scores calculated by each method. The highest-scaled scores are depicted in red, whereas the lowest scores are shown in blue. The top 40 genes are shown in the plot

To compare the genes identified in the IBD-PD cohort with genes known to be associated with IBD and PD, we combined genes with nominal significance (*P* < 0.01) from SKAT-O results and genes from significant NHC gene clusters into a single gene set (*n* = 120). We then compared it to the sets of known IBD (*n* = 157) and PD (*n* = 103) genes (Additional file 2: Table S8). Only *LRRK2* overlapped in known IBD and known PD gene sets. When comparing these gene sets with the IBD-PD-associated gene set, only 7 (5.83%) genes overlapped with known IBD genes and 4 (3.33%) genes overlapped with known PD genes (Additional file 2: Table S8). To evaluate the functional relevance of the 120 IBD-PD-associated genes to IBD and PD, we calculated their average biological distance (*D*_candidate_) to 157 known IBD genes and 103 known PD genes and found that *D*_candidate_ was 17.9 to known IBD genes whereas it was 19.2 to known PD genes (Fig. 3A). The average distances within the known IBD (*D*_IBD_) and PD (*D*_PD_) genes were 13.9 and 16.8, respectively. Then, we compared *D*_candidate_ to randomly sampled gene sets of equivalent size (*n* = 120) in 10,000 resampling iterations and obtained *P* values of 0.044 for IBD and 0.045 for PD, indicating that the biological relatedness of the candidate genes to IBD and PD was not random (Fig. 3A).

Pathway enrichment analysis with IPA showed that among the 104 significantly enriched pathways in the IBD-PD gene set, 6 pathways, “Iron homeostasis signaling pathway,” “Th1 Pathway,” “Autophagy,” “Th1 and Th2 Activation Pathway,” “Docosahexaenoic Acid (DHA) Signaling,” and “Th2 Pathway,” were significantly enriched in all three gene sets (Additional file 2: Table S9). A total of 75 significantly enriched pathways were shared between the IBD-PD and known IBD gene sets, primarily related to the inflammatory response and infections. By contrast, only 4 pathways were significantly enriched in both the IBD-PD and known PD gene sets, including those related to autophagy, endocytosis, and lysosomal biogenesis. Interestingly, 19 pathways were unique to the IBD-PD gene set including “Purine Nucleotides De Novo Biosynthesis II”, “Assembly of RNA Polymerase III Complex” and “Role of MAPK Signaling in Promoting the Pathogenesis of Influenza”. Additionally, 18 pathways were enriched in both known IBD and PD gene sets but not in the IBD-PD gene set, including “ERBB4 Signaling”, “Immunogenic Cell Death Signaling Pathway” and “Neuroinflammation Signaling Pathway”. These results suggested the potential role of inflammation and autophagy in the combined IBD-PD phenotype.

### Gene prioritization through biological relatedness, pathway, and gene enrichment analyses

To further identify and prioritize candidate genes associated with IBD-PD from the set of SKAT-O and NHC significant genes, we employed biological relatedness and pathway enrichment. As a result, 14 genes (*ATP11B*, *EPS15*, *IFNAR1*, *IL10RA*, *IL6ST*, *JAK1*, *LRRK2*, *NOD2*, *PIK3C3*, *PIKFYVE*, *RB1CC1*, *TRIM22*, *ULK2*, and *ZFYVE16*) were prioritized based on the overlapping genes from HumanBase, HGC, ToppGene and IPA results (Fig. 3B and Additional file 2: Table S10). Among 14 candidate genes, four (*IFNAR1*, *IL6ST*, *LRRK2*, and *NOD2*) were known IBD-associated genes [36], whereas *LRRK2* is also a well-known PD gene. We then explored HGMD for pathogenic mutations within candidate genes and found that 11 genes harbor “DM” (disease-causing) variants in various diseases (*EPS15* in congenital heart disease, *IFNAR1* and *JAK1* in immunodeficiency, *IL10RA* and *TRIM22* in IBD, *IL6ST* in hyper-IgE syndrome, *LRRK2* in PD, *NOD2* in CD, *PIKFYVE* in corneal dystrophy, *ULK2* in neurodevelopmental disorder and *ZFYVE16* in brain arteriovenous malformation).

In addition, we assessed the biological significance of the SKAT-O and NHC significant genes by calculating scores for shared interactions, pathways, and terms with known IBD and PD genes using PPI networks, pathway, and gene ontology enrichment analyses (Additional file 2: Tables S9, S11 and S12). The results revealed that genes involved in JAK-STAT and interferon alpha signaling, as well as autophagy-related genes, were particularly noteworthy as candidate genes for IBD-PD (Fig. 3C and Additional file 2: Table S13). Specifically, immunity-related genes, such as *JAK1*, *IL6ST*, *STAT2*, and *IFNAR1*, were more prominent in the IBD-specific analysis, whereas *LRRK2* and other phagosome-related genes including *PIK3C3*, *PIKFYVE*, *PIK3R4*, and *VAC14* were more significant in the PD-specific analysis (Additional file 1: Fig. S6, Additional file 2: Tables S14 and S15). Then, we investigated the tissue RNA expression patterns of the 14 prioritized genes using the consensus RNA tissue expression dataset from the Human Protein Atlas (https://www.proteinatlas.org/) [60]. We observed that 11 out of the 14 genes were ubiquitously expressed, whereas 3 exhibited tissue enrichment or enhancement in addition to being expressed in almost all tissues including the gastrointestinal tract, brain and bone marrow and lymphoid tissues: *LRRK2* showed tissue enrichment in the lung, *IL10RA* displayed tissue enhancement in the bone marrow and lymphoid tissue, and *NOD2* in bone marrow, esophagus, skin, and vagina. These findings further supported the potential role of these genes in the pathogenesis of IBD-PD comorbidity.

### PheWAS

**Fig. 4:**
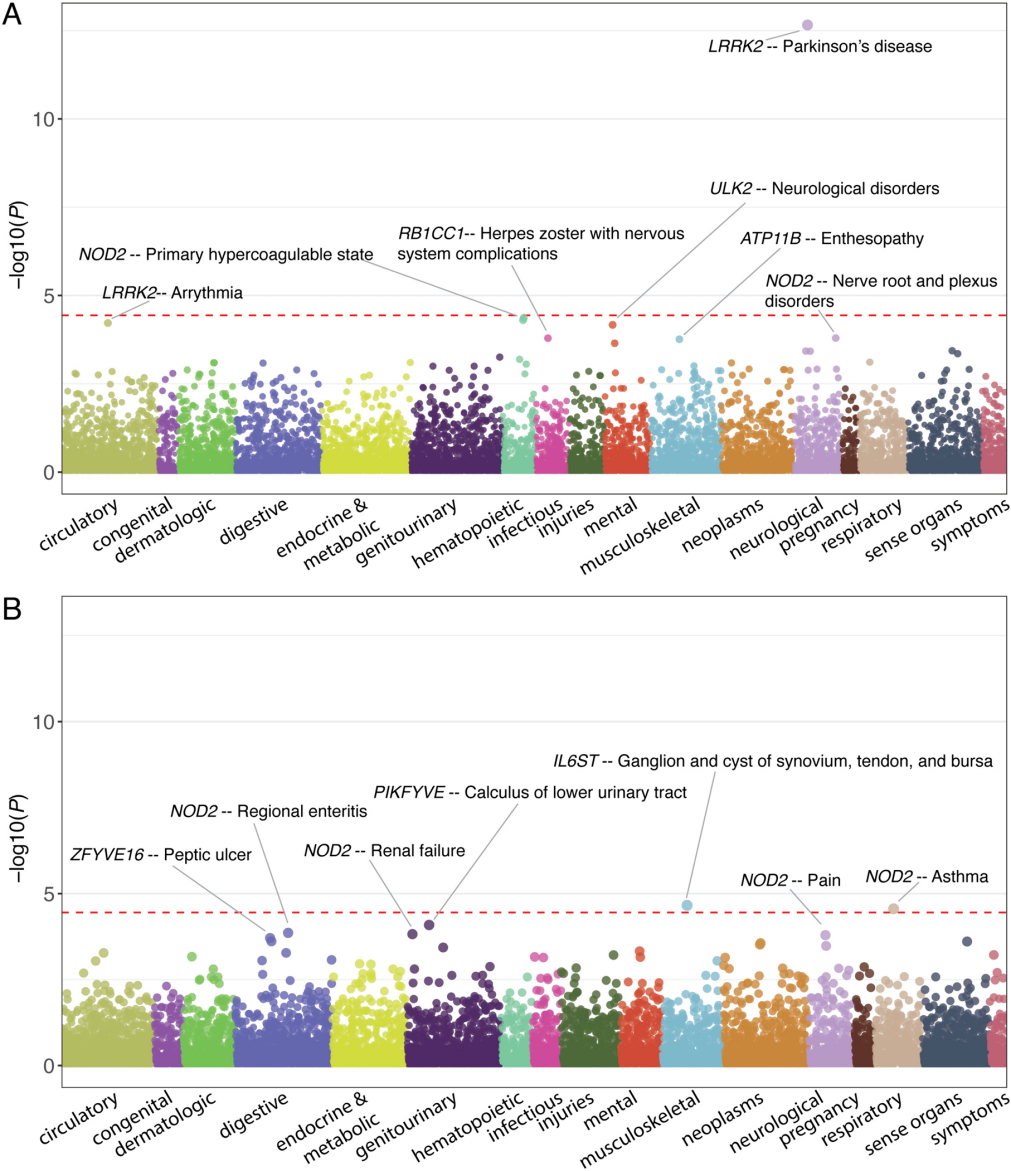
Gene-level PheWAS of 14 candidate genes in **A** Bio*Me* BioBank and **B** UK Biobank. The red dashed lines denote the study-wide significance levels

Lastly, we conducted a gene-level PheWAS in Bio*Me* BioBank and UK Biobank using presumably deleterious variants located in the 14 candidate genes that were identified in the IBD-PD cohort (Table S16) to validate the associations of candidate genes and explore their pleiotropic effects. The analysis in Bio*Me* BioBank revealed that the association between *LRRK2* and PD (phecode, 332) was the most significant result (*P* = 2.19 × 10^−13^), surpassing the phenome-wide significance level (Fig. 4A and Additional file 2: Table S17). Moreover, *LRRK2* was associated with torsion dystonia (333.4, *P* = 3.79 × 10^−4^) and “Extrapyramidal disease and abnormal movement disorders” (333, *P* = 1.29 × 10^−3^) in Bio*Me* BioBank. We further observed supporting results for the prioritized genes associated with IBD and PD in Bio*Me* with a nominal significance level. Specifically, *NOD2* was associated with CD (Regional enteritis, 555.1, *P* = 1.38 × 10^−4^ in UK Biobank and *P* = 2.56 × 10^−2^ in Bio*Me* BioBank) and IBD (*P* = 4.49 × 10^−2^ in UK Biobank and *P* = 2.29 × 10^−2^ in Bio*Me* BioBank) (Fig. 4B and Additional file 2: Table S18). *TRIM22* was associated with UC-related phecodes (556, *P* = 3.36 × 10^−3^, 578, *P* = 1.14 × 10^−2^, 556.1, *P* = 1.63 × 10^−2^ and 555.21, *P* = 2.89 × 10^−2^) and PD (332, *P* = 2.15 × 10^−2^) and “Myoclonus” (333.2, *P* = 2.51 × 10^−2^) in UK Biobank. It was also associated with “Extrapyramidal disease and abnormal movement disorders” and essential tremor in Bio*Me* BioBank (333, *P* = 1.79 × 10^−2^ and 333.1 2.87 × 10^−2^, respectively). *IL10RA* was found to be associated with UC (555.21 *P* = 2.09 × 10^−3^ in Bio*Me* and 3.69 × 10^−2^ in UK Biobank) and gastrointestinal hemorrhage (*P* = 1.67 × 10^−2^ in Bio*Me* and 1.67 × 10^−2^ in UK Biobank). Interestingly, *IL10RA* was also found to be associated with “Abnormality of gait” (350.2, *P* = 2.79 × 10^−2^) and “Abnormal movement” (350, *P* = 3.87 × 10^−2^) in Bio*Me* BioBank. *EPS15* exhibited associations with essential tremor (333.1, *P* = 1.03 × 10^−2^) in UK Biobank and “Abnormal movement” (350, *P* = 9.95 × 10^−3^) and “Abnormality of gait” (350.2, *P* = 3.99 × 10^−2^) in Bio*Me* BioBank. Furthermore, *PIKFYVE* showed an association with “Abnormal involuntary movements” in UK Biobank (350.1, *P* = 2.26 × 10^−2^), whereas it was associated with “Ulceration of intestine” (556.1, *P* = 2.91 × 10^−2^) and “Ulceration of the lower GI tract” (556, *P* = 4.67 × 10^−2^) in Bio*Me*. *ULK2* was associated with PD in UK Biobank (332, *P* = 3.5 × 10^−2^) and noninfectious gastroenteritis and “Hemorrhage of gastrointestinal tract” in Bio*Me* (*P* = 1.38 × 10^−2^ and 3.12 × 10^−2^). Additionally, *JAK1* was associated with essential tremor (333.1, *P* = 1.03 × 10^−2^) and *RB1CC1* displayed an association with cholangitis (575.1, *P* = 1.02 × 10^−2^) and *IL6ST* with noninfectious gastroenteritis in UK Biobank (558, *P* = 3.14 × 10^−2^). Lastly, we observed associations between *IFNAR1* and noninfectious gastroenteritis (558, *P* = 3.96 × 10^−3^), *ZFYVE16* and UC (555.21 *P* = 1.96 × 10^−2^), *ATP11B* and “Abnormality of gait” (350.2, *P* = 3.27 × 10^−2^), *PIK3C3* and PD and “Abnormal involuntary movements” (332, *P* = 3.49 × 10^−2^ and 350.1, *P* = 3.69 × 10^−2^) in Bio*Me* BioBank.

PheWAS results also revealed interesting pleiotropic effects of these genes, which have been shown to exert relevant functions in the identified phenotypes, such as the associations of *NOD2* and hypercoagulability (286.81 and 286.8) [61] and asthma (495) [62], *IL6ST* and joint disease (727.4) [63] and systemic and cutaneous lupus erythematosus (695.4, 695.42, and 695.41) [64], and *IFNAR1* and viral hepatitis [65]. Moreover, candidate genes displayed associations with various other autoimmune and allergic conditions, including ankylosing spondylitis, enthesopathy, rheumatoid arthritis, erythematous skin lesions, eosinophilia, and polyarteritis nodosa. These findings provide additional evidence for the functional significance of these genes in the development and manifestation of diverse phenotypes beyond the primary conditions of interest.

## Discussion

In the present study, we successfully replicated the well-established associations of the *LRRK2* G2019S and N2081D variants with IBD-PD. Both variants are located in the kinase domain of LRRK2 and have been linked to increased kinase activity [5, 48]. Furthermore, we identified 11 additional candidate *LRRK2* variants that may contribute to IBD-PD comorbidity. Previous studies have already reported L119P, S1228T, R1628P, M1646T, and Y2189C as PD risk variants [66–69], while I1371V has been shown to cause increased phosphorylation and aggregation of α-synuclein in neurons [70]. Additionally, P1542S is a common variant (gnomAD MAF = 0.03) and has been listed as a CD-associated polymorphism in HGMD [38]. E334K, R1325Q, and R1628P have been previously shown to increase the LRRK2- dependent Rab10 ^Thr73^ phosphorylation, which is used as an indicator of increased LRRK2 kinase activity conferring higher risk of PD [48]. This observation indicates that the carriers of these variants potentially benefit from LRRK2 kinase inhibitor therapy [48]. However, it is important to note that the remaining candidate *LRRK2* variants identified in the current study have been previously shown to have no impact on LRRK2-dependent rab10 ^Thr73^ phosphorylation, autophosphorylation of LRRK2^Ser1292^ or autophosphorylation of LRRK2^Ser935^. This suggests the possibility of the involvement of alternative mechanisms in the pathogenesis of IBD-PD comorbidity for these specific variants and further investigations are warranted to elucidate their precise effect.

On the other hand, we observed that the R1398H, N551K, and I723V variants were protective against IBD-PD but showed a trend to increase risk in the UC-specific and combined IBD analyses in Bio*Me* BioBank. The protective effect of the R1398H variant on PD and CD has been previously documented, which was linked to GTPase activation leading to a reduction in LRRK2 kinase activity. Also, N551K is in strong LD with R1398H [5, 38]. Our results may indicate no protective effect of these variants on UC, which probably contributed to the lack of association also observed in the combined IBD (CD + UC) analyses.

In the second part of the study, by examining the IBD-PD cohort using different computational approaches, we discovered both previously known and novel genes associated with PD and IBD. Although the sample size was relatively small, *LRRK2* and *IL10RA* attained genome-wide statistical significance in SKAT-O analysis and the *P* value of *DHRS2* was just below the Bonferroni-corrected significance threshold. IL10 plays a major role in anti-inflammatory processes, and variants in the genes encoding IL10 and IL10 receptors have been shown to be associated with very early onset IBD [71–74]. Impairments in IL10 production and signaling have also been implicated in neurodegenerative diseases [75] including PD [76–78]. *DHRS2* encodes a short-chain dehydrogenase/reductase that is involved in lipid metabolism and redox homeostasis. Under ischemic conditions, *DHRS2* exerts cytoprotective effects by reducing the accumulation of dicarbonyl compounds and reactive oxygen species (ROS) and implicated in the PD pathogenesis [79, 80].

As a complementary approach to SKAT-O, we utilized NHC, which is particularly advantageous for prioritizing candidate gene clusters in small cohorts for diseases with high genetic heterogeneity, like PD and IBD [49, 50], as conventional frequency-based case–control studies assume genetic homogeneity [34]. Through NHC analysis, we identified 6 biologically-relevant gene clusters, two of which contained previously known IBD- and/or PD-associated genes. We then examined significant genes obtained from SKAT-O analysis (*P* < 0.01) and genes within significant gene clusters from the NHC analyses. The results indicated that the average distance of these 120 significant genes to known IBD genes (*D*_candidate_ = 17.9) was slightly shorter than that to known PD genes (*D*_candidate_ = 19.2). However, when considering the shorter average biological distance within known IBD genes (*D*_IBD_ = 13.9) compared to that within known PD genes (*D*_PD_ = 16.8), these findings suggest similar contributions from IBD and PD to the IBD-PD-associated genes. Despite a minor overlap between IBD-PD-associated genes with known IBD and PD gene sets, the pathway enrichment results revealed a notable overlap in enriched pathways, especially between IBD and IBD-PD gene sets. In addition to 6 pathways related to autophagy and immune response enriched in all three gene sets, there were 10 pathways that were unique to IBD-PD-associated genes. Among these pathways were those involving three of the final 14 candidate genes, namely *PIK3C3*, *PIKFYVE*, and *JAK1*, along with several other genes. Of especial note, the genes involved in purine nucleotide biosynthesis, MAPK signaling, ephrin A signaling, LPS/IL-1 mediated inhibition of RXR function, and the NAD signaling pathway [81] could also be of particular interest for future studies, given their distinctive signatures for IBD-PD comorbidity, as well as their established or suggested roles in intestinal inflammation and neuronal metabolism [82–89]. By applying four distinct pathway enrichment and biological relatedness methods using both known IBD and known PD gene sets to reduce the likelihood of IBD-only or PD-only associations contributing to the observed comorbidity, we further identified and prioritized candidate genes associated with IBD-PD. This approach led us to identify 14 candidate genes, all of which have been reported to display functions that are relevant to IBD and PD pathogenesis (Additional file 1: Supplementary information regarding 14 prioritized genes). By calculating the biological importance scores using PPI networks and pathway and ontology enrichment, we showed that inflammation and autophagy-related genes likely play a significant role in IBD-PD pathogenesis.

The gene-level PheWAS in two different Biobanks further supported the PD and/or IBD associations of the candidate genes. Since we aimed to investigate the potential pathogenicity of the rare variants identified in the IBD-PD cohort, we exclusively used these variants to examine the associations of the candidate genes in the Bio*Me* BioBank and UK Biobank, which might have resulted in higher *P* values than anticipated when replicating some previously known associations. As expected, the most significant association was observed between *LRRK2* and PD. *TRIM22*, *IL10RA*, *PIKYVE*, and *ULK2* were found to be associated with both IBD- and PD-related phecodes, whereas *NOD2*, *RB1CC1*, *IL6ST*, *IFNAR1*, and *ZFYVE16* were associated specifically with IBD-related phecodes. Additionally, *LRRK2*, *JAK1*, *ATP11B*, and *PIK3C3* were associated with PD-related phecodes. Although not all candidate genes showed significant associations directly with IBD and PD diagnoses, we observed other relevant phenotypes that can be related to these diseases. For example, one of the earliest and most common motor changes observed in PD is the gait disturbance, which is caused by the impaired basal ganglia function [90]. Since *IL10RA*, *EPS15*, and *ATP11B* were associated with “Abnormality of gait,” they might be associated with PD. Further, *RB1CC1* was found to be associated with cholangitis, a disease closely associated with IBD [91]*.* These results support the potential connection between immune dysregulation, gut inflammation, and motor symptoms observed in PD and are in line with previous findings regarding the reduced incidence of PD in IBD patients receiving anti-inflammatory therapy [6, 9].

Previous studies investigating shared genetic factors between IBD and PD have mainly focused on common variants, leading to the identification of several loci associated with both diseases. Recently, a report examining the genetic correlation between IBD subtypes and PD using summary statistics of previous GWAS identified 23 novel loci in addition to the 9 loci reported previously [8]. Interestingly, *ATP6V0A1*, a gene that was identified in our NHC analysis, was among the novel pleiotropic loci shared between PD and CD and between PD and UC in that study. However, two recent Mendelian randomization studies, which also used summary statistics from large-scale GWAS of PD and IBD, failed to find a causal relationship between the two conditions [92, 93]. These conflicting findings might be attributable to the possibility that common genetic variants contributing to the IBD-PD comorbidity account for only a small fraction of the overall cases [50, 93]. Instead, the risks are driven by rarer variants. Other factors, such as chronic low-grade inflammation and environmental factors might cumulatively contribute to the development of PD in some IBD cases [6, 92].

However, in contrast to these previous studies that investigated the shared genetic factors between PD and IBD using separate cohorts with only one of these diseases phenotyped, our approach involved the analysis of WGS data from patients diagnosed with both IBD and PD. We used high-impact rare variants to identify novel genetic defects with larger effect sizes, which were not properly captured by GWAS studies due to their design. Applying our approach, we identified novel candidate genes implicated in inflammation and autophagy, which may play a role in the pathogenesis of IBD-PD comorbidity. However, it is important to consider the potential impact of incomplete penetrance, a common feature of many genetic diseases, including PD [94], on the manifestation of the disease in variant carriers. A thorough evaluation of the penetrance of our detected putative disease-causing variants can be more effectively performed in large case–control studies [95]. It will be crucial to continue investigating the genetic determinants of IBD-PD comorbidity to gain a comprehensive understanding of the underlying pathogenesis and develop effective risk stratification and personalized treatment strategies.

We believe that our study’s major strength is that, despite the limited sizes of the study cohorts to accommodate stringent variant-level analyses especially when considering rare variants, we applied a framework of analyses that were specifically designed to handle small sample sizes, thereby enabling novel gene discoveries for unique clinical phenotypes. It is nevertheless important to acknowledge certain limitations of our study. First, detailed clinical data were missing for the IBD-PD cohort owing to the strict regulation of the Danish Data Protection Agency for linking patient-level data with biospecimens, thereby preventing us from assessing the age of onset of these conditions or IBD subtypes (CD versus UC). Moreover, due to the lack of access to genetic data for IBD-only or PD-only patients, and healthy controls in the Danish Registry, we utilized existing datasets with available clinical information and performed extensive QC checks and genetic ancestry analyses to reduce the potential ascertainment bias. In addition, the disease cohorts were generated based on ICD codes, which may inherently lack granularity for certain disease subtypes. Also, we could not rule out genetic contributions from other immune-mediated diseases. Extensive evidence points towards a link of both IBD and PD with several organ-specific and multi-organ autoimmune disorders [96–101], making it challenging to exclude IBD and PD cases with concomitant immune-mediated diseases from the Bio*Me* BioBank and UK Biobank cohorts. Although adjusting association tests for polygenic risk scores for autoimmune diseases could reduce the potential confounding effect, there is a lack of a consensus on representative conditions that should be adjusted for. Moreover, such an approach might bias the results towards the null, especially those related to IBD, which itself is an immune-mediated condition. Nevertheless, an in-depth investigation of the potential confounding by autoimmune diseases is still warranted. An important aspect to consider when interpreting the results is that all IBD-PD cases included in this study share a common European ancestry and the results, therefore, may not be generalizable to more ethnically and racially diverse populations. Despite leveraging the ethnically-diverse Bio*Me* BioBank to validate our findings within the IBD-PD cohort, it is still crucial to validate these findings across diverse populations.

Furthermore, the relatively small cohorts used in the *LRRK2* coding sequence analysis limited our ability to observe and analyze very rare functional variants, as large sample sizes are pivotal for effectively detecting disease-associated rare variants and facilitating their replication across diverse cohorts [32]. Moreover, most of the *P* values in the variant-level analysis did not attain genome-wide statistical significance, which prevented us from drawing definitive conclusions. However, despite this limitation, we were able to replicate the well-known associations of the *LRRK2* G2019S and N2081D variants with both IBD and PD and identify potential novel causal variants. Furthermore, even though analyses to detect novel candidate genes associated with IBD-PD comorbidity were based on sequencing data from 67 IBD-PD cases, we used the NHC algorithm to address the low statistical power resulting from the small sample size and genetic heterogeneity, and identified six functionally-relevant gene clusters. In addition, by employing several pathway enrichment and biological relatedness approaches, we were able to prioritize genes from both SKAT-O and NHC results with high confidence. Lastly, the focus on high-impact rare variants during the discovery of novel candidate genes may have limited the detection of potential associations with common variants or variants with smaller effect sizes. Future studies incorporating rare and common variants, larger sample sizes in addition to analyses specific to the subtypes of IBD could provide a more comprehensive understanding of the genetic architecture underlying the IBD-PD comorbidity.

## Conclusions

In conclusion, our study highlights the significance of shared genetic factors in the IBD-PD overlap by both supporting previous findings and introducing novel candidate genes and variants. Future investigation of the interplay between inflammation and autophagy could in principle provide a better understanding of the shared etiology of IBD and PD and potential therapeutic targets for drug development and repurposing.

### Supplementary Information

**Additional file 1.** **Supplementary Information.** Supplementary Methods. Supplementary information regarding 14 prioritized genes. Supplementary references. **Fig. S1.** Cross-validation errors of the ADMIXTURE analysis of IBD-PD cases and the 1KGP populations. **Fig. S2.** ADMIXTURE analysis of IBD-PD cases and the 1KGP populations. **Fig. S3.** Scree plot of principal components. **Fig. S4.** QQ plots of the SKAT-O results. **Fig. S5.** STRING PPI network of the Cluster73 from NHC analysis. **Fig. S6.** Heatmap of the combined biological importance scores.

**Additional file 2.** **Table S1.** Sample-level QC metrics of the IBD**-**PD cohort. **Table S2.** Bio*Me* participants by ancestry. **Table S3.** Minor allele frequencies of the *LRRK2* variants. **Table S4.** Variant**-**level association tests of *LRRK2* variants. **Table S5.** HGMD classification of the LRRK2 missense variants. **Table S6.** SKAT**-**O results. **Table S7.** NHC results. **Table S8.** Known IBD and PD genes and 120 significant genes from SKAT-O and NHC analysis of IBD**-**PD cohort. **Table S9.** IPA canonical pathway enrichment analysis of 120 SKAT**-**O and NHC significant IBD**-**PD genes, known IBD genes and known PD genes. **Table S10.** Gene prioritization according to 4 biological relatedness and pathway approaches. **Table S11.** InnateDB pathway enrichment analysis. **Table S12.** InnateDB GO enrichment analysis. **Table S13.** Biological importance scores for IBD**-**PD. **Table S14.** Biological importance scores for PD. **Table S15.** Biological importance scores for IBD. **Table S16.** Presumably deleterious rare variants located in the 14 candidate genes identified in the IBD**-**PD cohort. **Table S17.** PheWAS of 14 candidate genes in Bio*Me* BioBank. **Table S18.** PheWAS of 14 candidate genes in UK Biobank.

## Data Availability

The WGS data of the IBD-PD cohort and the WES data of the Bio*Me* BioBank cohorts used and/or analyzed in this study are available from the corresponding authors upon reasonable request within one week. This research was conducted using the UK Biobank resource under application number 53074. UK Biobank WES datasets are accessible upon approval of application. The 1KGP WGS data used in the current study is available at http://ftp.1000genomes.ebi.ac.uk/vol1/ftp/data_collections/1000G_2504_high_coverage/working/20190425_NYGC_GATK/raw_calls_updated/ [18]. The gene- and variant-level association statistics generated in this study are provided in the supplementary data. The Mutation Significance Cutoff and the Gene Damage Index databases for selecting high-impact variants and genes are available at https://lab.rockefeller.edu/casanova/MSC [28] and https://lab.rockefeller.edu/casanova/GDI [29]. The Human Gene Connectome is available for download at https://lab.rockefeller.edu/casanova/HGC [39]. Other tools and methods used in the analysis are described in the Methods section with references.

## Abbreviations

1KGP: 1000 Genomes Project Phase 3
CD: Crohn’s disease
DM: Disease-causing mutation
EHR: Electronic health records
GATK: Genome Analysis Toolkit
GDI: Gene Damage Index
GO: Gene Ontology
HGC: Human Gene Connectome
HGMD: Human Gene Mutation Database
HWE: Hardy-Weinberg equilibrium
IBD: Inflammatory bowel disease
IPA: Ingenuity Pathway Analysis
LD: Linkage disequilibrium
MAC: Minor allele count
MAF: Minor allele frequency
MSC: Mutation Significance Cutoff
NHC: Network-based heterogeneity clustering
OR: Odds ratio
PC: Principal component
PCA: Principal component analysis
PD: Parkinson’s disease
PheWAS: Phenome-wide association studies
QC: Quality control
SKAT**-**O: Optimized sequence kernel association test
UC: Ulcerative colitis
VEP: Variant effect predictor
WES: Whole exome sequencing
WGS: Whole genome sequencing
