# Supplementary material for: The landscape of rare genetic variation associated with inflammatory bowel disease and Parkinson’s disease comorbidity
Source: Genome Med. 2024 May 14;16:66. doi: 10.1186/s13073-024-01335-2 (PMC11092054; doi:10.1186/s13073-024-01335-2)
Supplement: Supplementary file 1 — Additional file 1. Supplementary Information. Supplementary Methods. Supplementary information regarding 14 prioritized genes. Supplementary references. Fig. S1. Cross-validation errors of the ADMIXTURE analysis of IBD-PD cases and the 1KGP populations. Fig. S2. ADMIXTURE analysis of IBD-PD cases and the 1KGP populations. Fig. S3. Scree plot of principal components. Fig. S4. QQ plots of the SKAT-O results. Fig. S5. STRING PPI network of the Cluster73 from NHC analysis. Fig. S6. Heatmap of the combined biological importance scores. [file 13073_2024_1335_MOESM1_ESM.docx]

**Supplementary Information**

**The landscape of rare genetic variation associated with inflammatory bowel disease and Parkinson's disease comorbidity**

Meltem Ece Kars^1^, Yiming Wu^1,2^, Peter D. Stenson^3^, David N. Cooper^3^, Johan Burisch,^4,5,6^ Inga Peter^7,&,*^, Yuval Itan^1,7,8,&,*^

^1^The Charles Bronfman Institute for Personalized Medicine, Icahn School of Medicine at Mount Sinai, New York, NY 10029, USA

^2^College of Life Science, China West Normal University, Nan Chong, Si Chuan 637009, China

^3^Institute of Medical Genetics, Cardiff University, Cardiff, CF14 4XN, UK

^4^Gastrounit, Medical Division, Copenhagen University Hospital – Amager and Hvidovre, Kettegård Alle 30, Hvidovre 2650, Copenhagen, Denmark

^5^Copenhagen Center for Inflammatory Bowel Disease in Children, Adolescents and Adults, Copenhagen University Hospital – Amager and Hvidovre, Kettegård Alle 30, Hvidovre 2650, Copenhagen, Denmark

^6^Department of Clinical Medicine, Faculty of Health and Medical Sciences, University of Copenhagen, Blegdamsvej 3B, Copenhagen 2200, Denmark

^7^Department of Genetics and Genomic Sciences, Icahn School of Medicine at Mount Sinai, New York, NY 10029, USA

^8^Mindich Child Health and Development Institute, Icahn School of Medicine at Mount Sinai, New York, NY 10029, USA.

^&^Equal contribution

*Corresponding authors.

Yuval Itan: [yuval.itan@mssm.edu](mailto:yuval.itan@mssm.edu),

Inga Peter: [inga.peter@mssm.edu](mailto:inga.peter@mssm.edu)

**Contents**

[**Supplementary Methods** 2](#_Toc163826144)

[**Identification of the IBD-PD samples in the Danish National Biobank** 2](#_Toc163826145)

[**Whole genome sequencing of the IBD-PD samples** 2](#_Toc163826146)

[**Genotyping and quality control of the IBD-PD cohort** 3](#_Toc163826147)

[**Determining the genetic ancestries of the IBD-PD cases** 3](#_Toc163826148)

[**Identification of IBD and PD samples with European descent from Bio*Me* and UK Biobank** 3](#_Toc163826149)

[**Methods used in biological relatedness, pathway and gene enrichment analysis** 4](#_Toc163826150)

[**Supplementary information regarding 14 prioritized genes** 5](#_Toc163826151)

[**Supplementary References** 5](#_Toc163826152)

[**Fig. S1.** Cross-validation errors of the ADMIXTURE analysis of IBD-PD cases and the 1KGP populations. 8](#_Toc163826153)

[**Fig. S2.** ADMIXTURE analysis of IBD-PD cases and the 1KGP populations. 8](#_Toc163826154)

[**Fig. S3.** Scree plot of principal components. 9](#_Toc163826155)

[**Fig. S4.** QQ plots of the SKAT-O results. 10](#_Toc163826156)

[**Fig. S5.** STRING PPI network of the Cluster73 from NHC analysis. 11](#_Toc163826157)

[**Fig. S6.** Heatmap of the combined biological importance scores. 12](#_Toc163826158)

# **Supplementary Methods**

## **Identification of the IBD-PD samples in the Danish National Biobank**

In Denmark, every resident is assigned a ten-digit identification number either at birth or upon immigration through the Civil Registration System. This system provides basic demographic data such as sex, date of birth, date of death or emigration and is linked to the Danish National Patient registry (NPR). The NPR comprises all hospitalization records since 1977 and all outpatient visits, medical procedures, and surgical interventions since 1995. Using diagnosis codes, we identified all patients diagnosed with both IBD (using ICD-8 codes 563.01, 563.19, 569.04 and ICD-10 codes K50, K51) and PD (using ICD-8 code 342 and ICD-10 code G20) within the NPR. This methodology to identify patients with both IBD and PD has been shown in previous studies to have high validity [1, 2]. Subsequently, we linked this cohort of patients with both IBD and PD diagnoses (IBD-PD cohort) with the Danish National Biobank resource (<https://www.danishnationalbiobank.com/>), which links nationwide Danish registries with biosamples in Danish biobanks. This process allowed us to identify 76 IBD-PD patients whose whole blood samples were stored in the Copenhagen Hospital Biobank [3]. This biobank has been established from surplus whole blood obtained from patients admitted to Danish hospitals in the Copenhagen region for diagnostic or treatment purposes where blood samples have been drawn for blood type testing or red cell antibody screening. Whole blood (2x850 µL) from these patients was then retrieved from the biobank and included in the present study.

## **Whole genome sequencing of the IBD-PD samples**

Whole-genome sequencing (WGS) was performed for a total of 76 IBD-PD cases using the Illumina HiSeq X platform with 2x150 bp paired-end reads. The raw reads were aligned to the GRCh38 reference genome using Burrows-Wheeler Aligner (BWA) [4]. Duplicate reads were removed, and recalibration was performed using Picard tools (<http://broadinstitute.github.io/picard/>). Sample-level genotype variant calling format (gVCF) files were generated using HaplotypeCaller, following the best practice guidelines of Genome Analysis Toolkit (GATK) [5].

## **Genotyping and quality control of the IBD-PD cohort**

Joint genotyping of the gVCF files of the 76 IBD-PD samples and the 427 1KGP samples was performed using GATK v.3.7. Insertion-deletion variants (indel) normalization and splitting of multi-allelic variants were carried out using BCFtools [6]. Variant quality score recalibration (VQSR) was performed using GATK, with tranche sensitivities set to 99.5 for single nucleotide polymorphisms (SNPs) and 99.0 for short indels. Variants with a "PASS" filter status were retained for further analysis. Genotypes with a depth less than 8 and a genotype quality below 20 were set to missing. Variants with a missingness rate exceeding 20% and those located in low-complexity regions (LCRs) were filtered out. LCRs were identified using the RepeatMasker track of UCSC table browser (<https://genome.ucsc.edu/cgi-bin/hgTables>). Furthermore, variants with a high differential missingness rate (>10%) between cases and controls were excluded from the analysis.

Biological sex was determined using PLINK v.1.9 [7]. Sample-level quality control (QC) was conducted using autosomal variants and BCFtools stats by following a similar approach to that of gnomAD v.3.1 [8]. Samples were excluded if they fell outside of 5 standard deviations (SD) from the mean for the following metrics: number of deletions, number of insertions, insertion-deletion ratio, number of heterozygous variants, number of homozygous variants for the alternate allele, number of transitions (Ti), number of transversions (Tv), Ti-Tv ratio, and number of SNPs. Additionally, samples were excluded if the number of singletons or the heterozygote-homozygote ratio exceeded the mean by 5 SD. As a result of sample-level QC, four samples were excluded (Additional file 2: Table S1).

KING analysis to detect second-degree or closer relatives revealed a pair of full siblings within the 1KGP control group, which led to removal of one control sample from the dataset [9]. Additionally, five IBD-PD samples were excluded from the analysis due to their contribution to inflated *P* values in SKAT-O with synonymous variants (refer to “SKAT-O and Network-based heterogeneity clustering of the IBD-PD cohort”). The final dataset comprised a total of 20,158,023 variants across 67 IBD-PD cases and 426 controls. 40 (59.7%) IBD-PD samples and 214 (50.2%) control samples were males.

## **Determining the genetic ancestries of the IBD-PD cases**

To ascertain the genetic ancestries of the IBD-PD cases, we conducted a principal component analysis (PCA) utilizing 1KGP populations as a reference. We first combined the WGS data from the 67 IBD-PD cases and 2,548 samples from the 1KGP [10]. The analysis was performed using PLINK v1.9 with linkage disequilibrium (LD)-pruned variants (*r*^2^ = 0.2) with a minor allele frequency (MAF) greater than 5% and not exceeding Hardy-Weinberg equilibrium (HWE) with a *P* < 1x10^-6^ [7].

We also conducted an ADMIXTURE analysis using the same variants included in the PCA [11]. ADMIXTURE was run for the *K* from 2 to 10 where *K* = 8 revealed the lowest cross-validation error. Based on the results of the analysis with *K*=8, the genetic ancestries of all IBD-PD cases were identified as European. Specifically, 52 cases exhibited ancestral components similar to Northwestern European populations (CEU and GBR), while 15 cases displayed a closer resemblance to Southern European populations (IBS and TSI).

## **Identification of IBD and PD samples with European descent from Bio*Me* and UK Biobank**

In both Bio*M*e BioBank and UK Biobank, individuals of European descent were genetically determined by the following steps: first, autosomal variants from biobank participants were merged with those from Utah residents (CEPH) with Northern and Western European ancestry (CEU), Han Chinese in Beijing, China (CHB), Japanese in Tokyo, Japan (JPT) and Yoruba in Ibadan, Nigeria (YRI) populations of HapMap3 [12]. Then, variants were pruned according to LD using an *r*^2^ = 0.2 threshold and filtered for those with a MAF > 2%. Admixture analysis was conducted using fastSTRUCTURE [13] with a K = 3 to represent individuals with African, East Asian and European ancestries. Subsequently, individuals with a European ancestry fraction greater than 0.8 were retained.

Cases and controls were identified based on ICD9 and ICD10 diagnoses of Bio*Me* BioBank and UK Biobank participants. For defining CD cases, diagnostic codes 555.* (ICD-9) and K50.* (ICD-10) were used, and for defining UC cases, diagnostic codes 556.* (ICD-9) and K51.* diagnosis codes were used. The IBD cohort included both CD and UC cases. Instances where individuals exhibited both CD and UC-related ICD codes were excluded from both case groups but were included within the IBD cohort (*n* = 37 for Bio*Me* BioBank and *n* = 196 for UK Biobank). PD cases were defined by the presence of codes 332.0 (ICD-9) and G20 (ICD10). Individuals diagnosed with both IBD and PD were removed from the dataset, as their number was insufficient for conducting a separate IBD-PD analysis (*n* = 6 in Bio*Me* BioBank and *n* = 13 in UK Biobank). The control groups were generated by randomly selecting 800 (Bio*Me* BioBank) and 3,000 (UK Biobank) European participants who had not received diagnoses for any gastrointestinal disease (ICD-9: 520-579 and ICD-10: K*) or neurological disease (ICD-9: 320-359 and ICD-10: G*).

## **Methods used in biological relatedness, pathway and gene enrichment analysis**

*ToppGene*

We utilized ToppGene to prioritize candidate genes by evaluating their functional similarity to known IBD and PD genes [14]. The default training parameters of ToppGene, which include Gene Ontology (GO) terms, pathways, diseases, and PubMed records, were employed. Candidate genes with a Benjamini-Hochberg (BH)-adjusted *P* value of less than 0.05 were retained.

*Ingenuity pathway analysis (IPA)*

We performed Ingenuity Pathway Analysis (IPA) network analysis (QIAGEN Inc., version 01-21-03

<https://www.qiagenbioinformatics.com/products/ingenuity-pathway-analysis>) on 120 candidate genes. Specifically, we selected causal networks related to PD and inflammation of the gastrointestinal tract to calculate the network scores. Candidate genes were prioritized based on the identified networks associated with ‘Cell Signaling, Post-Translational Modification, Protein Synthesis’ (network score = 67) and ‘Cell Cycle, Cell Death and Survival, Cellular Growth and Proliferation’ (network score = 55).

*HumanBase*

We utilized HumanBase for functional module discovery, specifically employing the global network option [15]. Gene clusters identified within the functional modules were then utilized to prioritize IBD-PD candidate genes.

*The human gene connectome*

We employed the human gene connectome (HGC) to detect candidate genes that have the shortest biological distances to known IBD and PD genes [16]. For each candidate gene, we calculated the average distances to all known IBD and PD genes (*D*_candidate_) as we previously described [17]. Additionally, we calculated the average distances within the known IBD and PD genes (*D*_IBD_ and *D*_PD_). We retained genes with a *D*_candidate_ value lower than both *D*_IBD_ and *D*_PD_ as prioritized IBD-PD candidates.

Furthermore, we conducted resampling tests to demonstrate that the candidate gene set has a shorter average biological distance to known IBD and PD genes compared to randomly selected genes (*D*_random_). Resampling was performed through 10,000 iterations using random gene sets containing 120 genes, which were equivalent in size to the candidate gene set. The number of random sets that showed a shorter average distance to known genes than the candidate genes (*D*_random_ < *D*_candidate_) was determined to calculate an empirical *P* value.

# **Supplementary information regarding 14 prioritized genes**

The candidate genes identified in this study are primarily related to autophagy and inflammation, two interconnected mechanisms, suggesting a potential shared pathogenic mechanism between the two conditions [18]. *ATP11B* encodes a member of the P4-type phospholipid transferase family and is involved in immunity and signal transduction [19]. It is implicated in the gut-brain axis, as its deletion in mice resulted in alterations in gut microbiota and accelerated brain aging [20]. *ATP11B* deficiency has also been associated with synaptic dysfunction, which is linked to PD [21]. *EPS15* is another interesting candidate, functioning in epidermal growth factor receptor (EGFR) trafficking and signaling together with parkin, thereby linked it to PD [22]. Considering the role of EGFR in maintaining mucosal and epithelial barrier integrity of gastrointestinal tract, *EPS15* is a plausible candidate for IBD-PD [23]. *IFNAR1*, *IL10RA*, *IL6ST* and *JAK1* are involved in the JAK-STAT signaling, which is targeted in the treatment of malignancies, immune-mediated diseases including IBD, and PD [24]. Besides, several genes in this pathway are associated with IBD [25]. Moreover, deficiency of neuronal IFN-β-IFNAR has been linked to Lewy body- and PD-like dementia, indicating the role of inflammation in the pathogenesis of neurodegeneration [26].

*NOD2*, *PIK3C3*, *PIKFYVE*, *RB1CC1* and *ULK2* are involved in autophagosome assembly and endosomal activity. Reduced autophagy leading to accumulation of protein aggregates and subsequent cell degeneration is a pathological hallmark of PD [18]. Moreover, autophagy contributes to pathogenesis and progression of both CD and UC. Several autophagy-related genes were identified in GWAS of CD, including *NOD2*, *ATG16L1* and *ULK1* [27]. *LRRK2* variants have been shown to alter autophagy-lysosome response to cellular stress thereby contributing to the pathogenesis of CD [28]. *NOD2* is a well-established CD-associated gene and has also been found to contribute to dopaminergic neuron degeneration [25, 29]. Consistently, a previous GWAS identified an association between the p.G908R variant in *NOD2* and PD [30]. *PIK3C3* is another autophagy-related gene whose function has been implicated in PD and IBD. Studies have shown that the p.R768W variant in *PIK3C3* is associated with PD [31], and *PIK3C3* knockout in zebrafish resulted in IBD [32].

*TRIM22* and *ZFYVE16* are two other candidate genes that were prioritized in this study. *TRIM22* encodes an antiviral factor induced by interferons that interacts with NOD2 signaling. Variants in *TRIM22* have been shown to be associated with very early onset IBD due to increased inflammation [33]. *ZFYVE16* encodes an endosomal protein that regulates endosomal trafficking, thereby facilitating transforming growth factor-β (TGF- β) signaling. Dysregulated TGF- β is a key player in intestinal fibrosis in IBD [34]. Furthermore, impaired neuronal TGF- β signaling causes neurodegeneration in the nigrostriatal system in mice [35].

# **Supplementary References**

1. Fonager K, Sorensen HT, Rasmussen SN, Moller-Petersen J, Vyberg M. Assessment of the diagnoses of Crohn's disease and ulcerative colitis in a Danish hospital information system. Scand J Gastroenterol. 1996;31(2):154-9.

2. Wermuth L, Lassen CF, Himmerslev L, Olsen J, Ritz B. Validation of hospital register-based diagnosis of Parkinson's disease. Dan Med J. 2012;59(3):A4391.

3. Sorensen E, Christiansen L, Wilkowski B, Larsen MH, Burgdorf KS, Thorner LW, et al. Data resource profile: The Copenhagen Hospital Biobank (CHB). Int J Epidemiol. 2021;50(3):719-20e.

4. Li H, Durbin R. Fast and accurate short read alignment with Burrows-Wheeler transform. Bioinformatics. 2009;25(14):1754-60.

5. Van der Auwera GA, Carneiro MO, Hartl C, Poplin R, Del Angel G, Levy-Moonshine A, et al. From FastQ data to high confidence variant calls: the Genome Analysis Toolkit best practices pipeline. Curr Protoc Bioinformatics. 2013;43(1110):11.0.1-.0.33.

6. Danecek P, Bonfield JK, Liddle J, Marshall J, Ohan V, Pollard MO, et al. Twelve years of SAMtools and BCFtools. Gigascience. 2021;10(2):giab008.

7. Purcell S, Neale B, Todd-Brown K, Thomas L, Ferreira MA, Bender D, et al. PLINK: a tool set for whole-genome association and population-based linkage analyses. Am J Hum Genet. 2007;81(3):559-75.

8. Karczewski KJ, Francioli LC, Tiao G, Cummings BB, Alföldi J, Wang Q, et al. The mutational constraint spectrum quantified from variation in 141,456 humans. Nature. 2020;581(7809):434-43.

9. Manichaikul A, Mychaleckyj JC, Rich SS, Daly K, Sale M, Chen WM. Robust relationship inference in genome-wide association studies. Bioinformatics. 2010;26(22):2867-73.

10. Auton A, Brooks LD, Durbin RM, Garrison EP, Kang HM, Korbel JO, et al. A global reference for human genetic variation. Nature. 2015;526(7571):68-74.

11. Alexander DH, Novembre J, Lange K. Fast model-based estimation of ancestry in unrelated individuals. Genome Res. 2009;19(9):1655-64.

12. International HapMap Consortium, Altshuler DM, Gibbs RA, Peltonen L, Altshuler DM, Gibbs RA, et al. Integrating common and rare genetic variation in diverse human populations. Nature. 2010;467(7311):52-8.

13. Raj A, Stephens M, Pritchard JK. fastSTRUCTURE: variational inference of population structure in large SNP data sets. Genetics. 2014;197(2):573-89.

14. Chen J, Bardes EE, Aronow BJ, Jegga AG. ToppGene Suite for gene list enrichment analysis and candidate gene prioritization. Nucleic Acids Res. 2009;37(Web Server issue):W305-11.

15. Greene CS, Krishnan A, Wong AK, Ricciotti E, Zelaya RA, Himmelstein DS, et al. Understanding multicellular function and disease with human tissue-specific networks. Nat Genet. 2015;47(6):569-76.

16. Itan Y, Zhang SY, Vogt G, Abhyankar A, Herman M, Nitschke P, et al. The human gene connectome as a map of short cuts for morbid allele discovery. Proc Natl Acad Sci U S A. 2013;110(14):5558-63.

17. Wu Y, Gettler K, Kars ME, Giri M, Li D, Bayrak CS, et al. Identifying high-impact variants and genes in exomes of Ashkenazi Jewish inflammatory bowel disease patients. Nat Commun. 2023;14(1):2256.

18. Minchev D, Kazakova M, Sarafian V. Neuroinflammation and autophagy in Parkinson's disease-novel perspectives. Int J Mol Sci. 2022;23(23):14997.

19. Liu C, Zhang S, Shi H, Zhou H, Zhuang J, Cao Y, et al. *Atp11b* deletion affects the gut microbiota and accelerates brain aging in mice. Brain Sci. 2022;12(6):709.

20. Arimoto T, Choi DY, Lu X, Liu M, Nguyen XV, Zheng N, et al. Interleukin-10 protects against inflammation-mediated degeneration of dopaminergic neurons in substantia nigra. Neurobiol Aging. 2007;28(6):894-906.

21. Wang J, Li W, Zhou F, Feng R, Wang F, Zhang S, et al. *ATP11B* deficiency leads to impairment of hippocampal synaptic plasticity. J Mol Cell Biol. 2019;11(8):688-702.

22. van Bergen En Henegouwen PM. Eps15: a multifunctional adaptor protein regulating intracellular trafficking. Cell Commun Signal. 2009;7:24.

23. Brandl K, Sun L, Neppl C, Siggs OM, Le Gall SM, Tomisato W, et al. MyD88 signaling in nonhematopoietic cells protects mice against induced colitis by regulating specific EGF receptor ligands. Proc Natl Acad Sci U S A. 2010;107(46):19967-72.

24. Hu X, Li J, Fu M, Zhao X, Wang W. The JAK/STAT signaling pathway: from bench to clinic. Signal Transduct Target Ther. 2021;6(1):402.

25. Jostins L, Ripke S, Weersma RK, Duerr RH, McGovern DP, Hui KY, et al. Host-microbe interactions have shaped the genetic architecture of inflammatory bowel disease. Nature. 2012;491(7422):119-24.

26. Ejlerskov P, Hultberg JG, Wang J, Carlsson R, Ambjorn M, Kuss M, et al. Lack of neuronal IFN-beta-IFNAR causes Lewy body- and Parkinson's disease-like dementia. Cell. 2015;163(2):324-39.

27. Shao BZ, Yao Y, Zhai JS, Zhu JH, Li JP, Wu K. The role of autophagy in inflammatory bowel disease. Front Physiol. 2021;12:621132.

28. Hui KY, Fernandez-Hernandez H, Hu J, Schaffner A, Pankratz N, Hsu NY, et al. Functional variants in the *LRRK2* gene confer shared effects on risk for Crohn's disease and Parkinson's disease. Sci Transl Med. 2018;10(423):eaai7795.

29. Cheng L, Chen L, Wei X, Wang Y, Ren Z, Zeng S, et al. NOD2 promotes dopaminergic degeneration regulated by NADPH oxidase 2 in 6-hydroxydopamine model of Parkinson's disease. J Neuroinflammation. 2018;15(1):243.

30. Germer EL, Imhoff S, Vilarino-Guell C, Kasten M, Seibler P, Bruggemann N, et al. The role of rare coding variants in Parkinson's disease GWAS Loci. Front Neurol. 2019;10:1284.

31. Goldstein O, Gana-Weisz M, Banfi S, Nigro V, Bar-Shira A, Thaler A, et al. Novel variants in genes related to vesicle-mediated-transport modify Parkinson's disease risk. Mol Genet Metab. 2023;139(2):107608.

32. Zhao S, Xia J, Wu X, Zhang L, Wang P, Wang H, et al. Deficiency in class III PI3-kinase confers postnatal lethality with IBD-like features in zebrafish. Nat Commun. 2018;9(1):2639.

33. Li Q, Lee CH, Peters LA, Mastropaolo LA, Thoeni C, Elkadri A, et al. Variants in *TRIM22* that affect NOD2 signaling are associated with very-early-onset inflammatory bowel disease. Gastroenterology. 2016;150(5):1196-207.

34. Ihara S, Hirata Y, Koike K. TGF-beta in inflammatory bowel disease: a key regulator of immune cells, epithelium, and the intestinal microbiota. J Gastroenterol. 2017;52(7):777-87.

35. Tesseur I, Nguyen A, Chang B, Li L, Woodling NS, Wyss-Coray T, et al. Deficiency in neuronal TGF-beta signaling leads to nigrostriatal degeneration and activation of TGF-beta signaling protects against MPTP neurotoxicity in mice. J Neurosci. 2017;37(17):4584-92.

# **Fig. S1.** Cross-validation errors of the ADMIXTURE analysis of IBD-PD cases and the 1KGP populations.


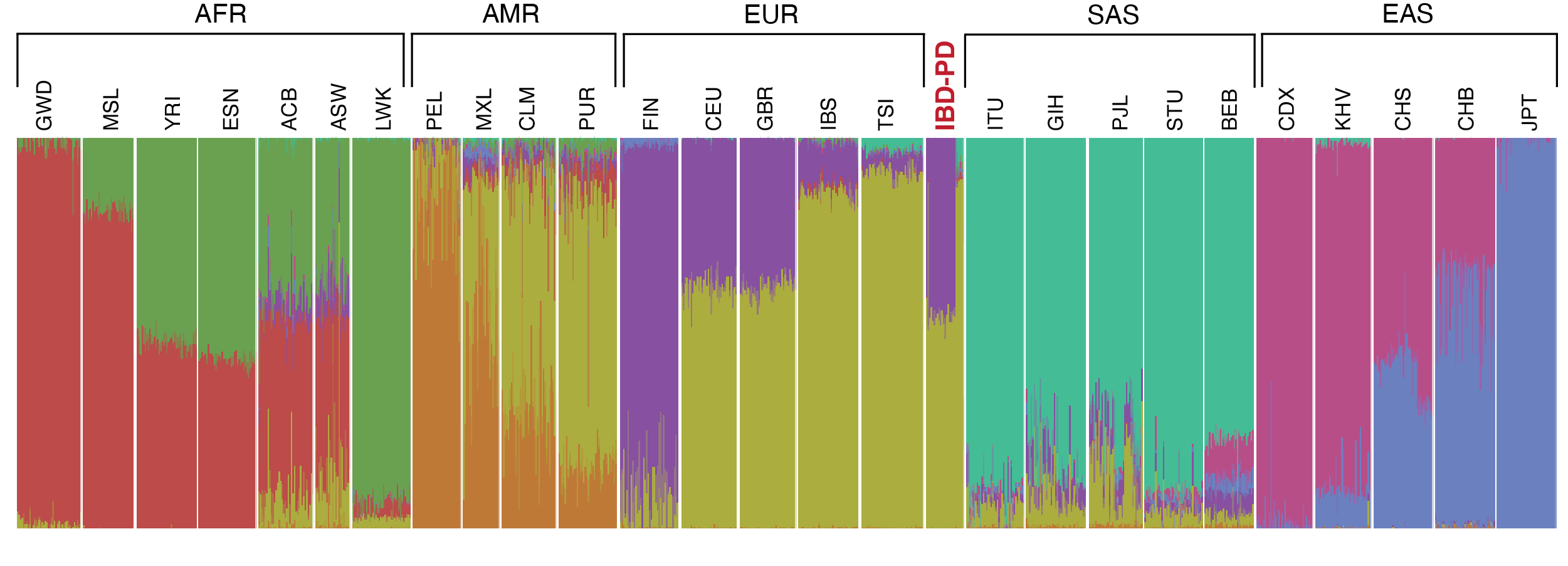


# **Fig. S2.** ADMIXTURE analysis of IBD-PD cases and the 1KGP populations.

67 IBD-PD cases and 2,548 samples from 1KGP were included. The plot was generated using *K*=8, determined as the most optimal number of ancestral components by ADMIXTURE. Each vertical bar represents the ancestral proportions of a single individual. Descriptions of the 1KGP subpopulations can be obtained from the International Genome Sample Resource: <https://www.internationalgenome.org/data-portal/population>.


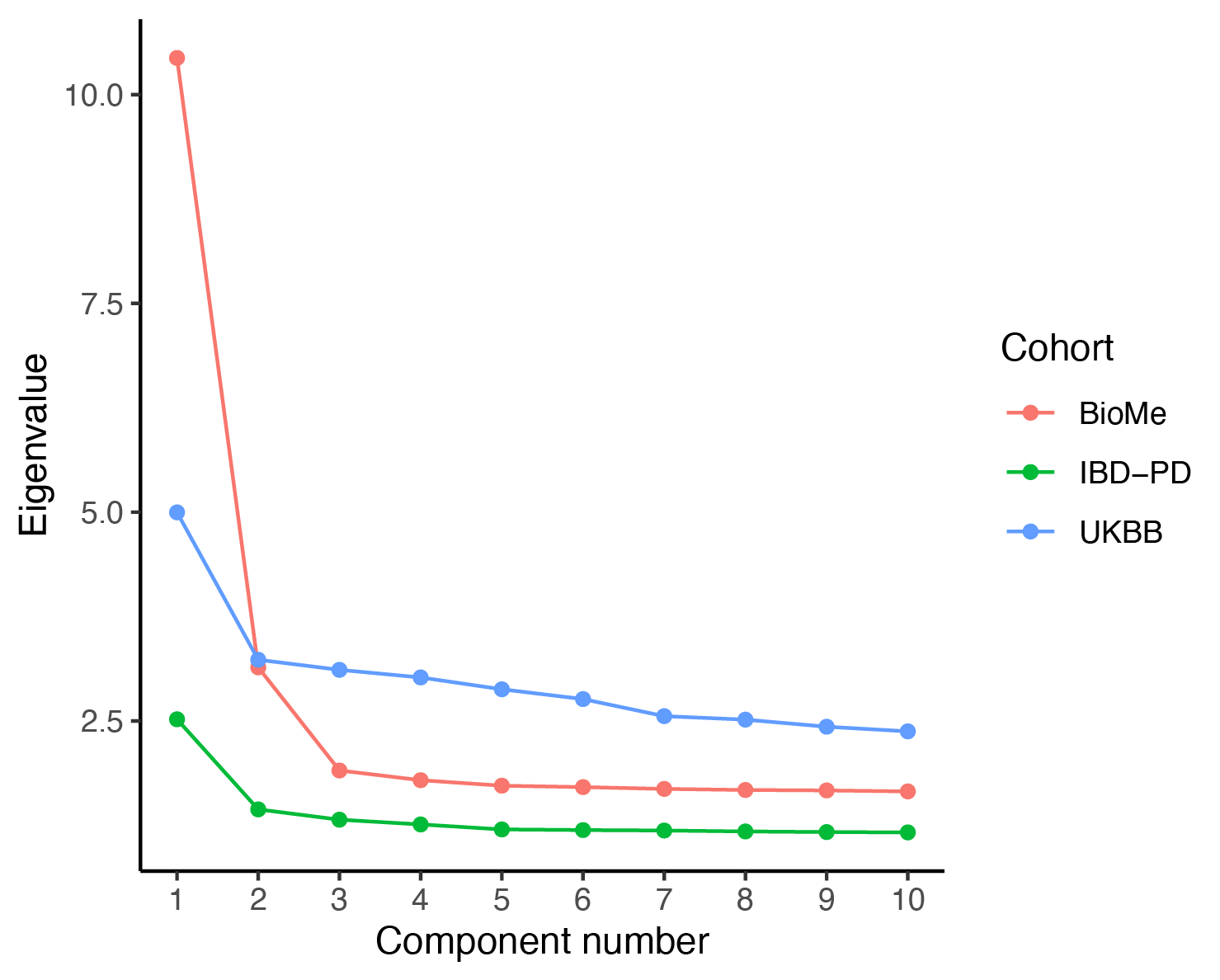


# **Fig. S3.** Scree plot of principal components.

Eigenvalue Scree plot depicting the principal component eigenvalues for each cohort.


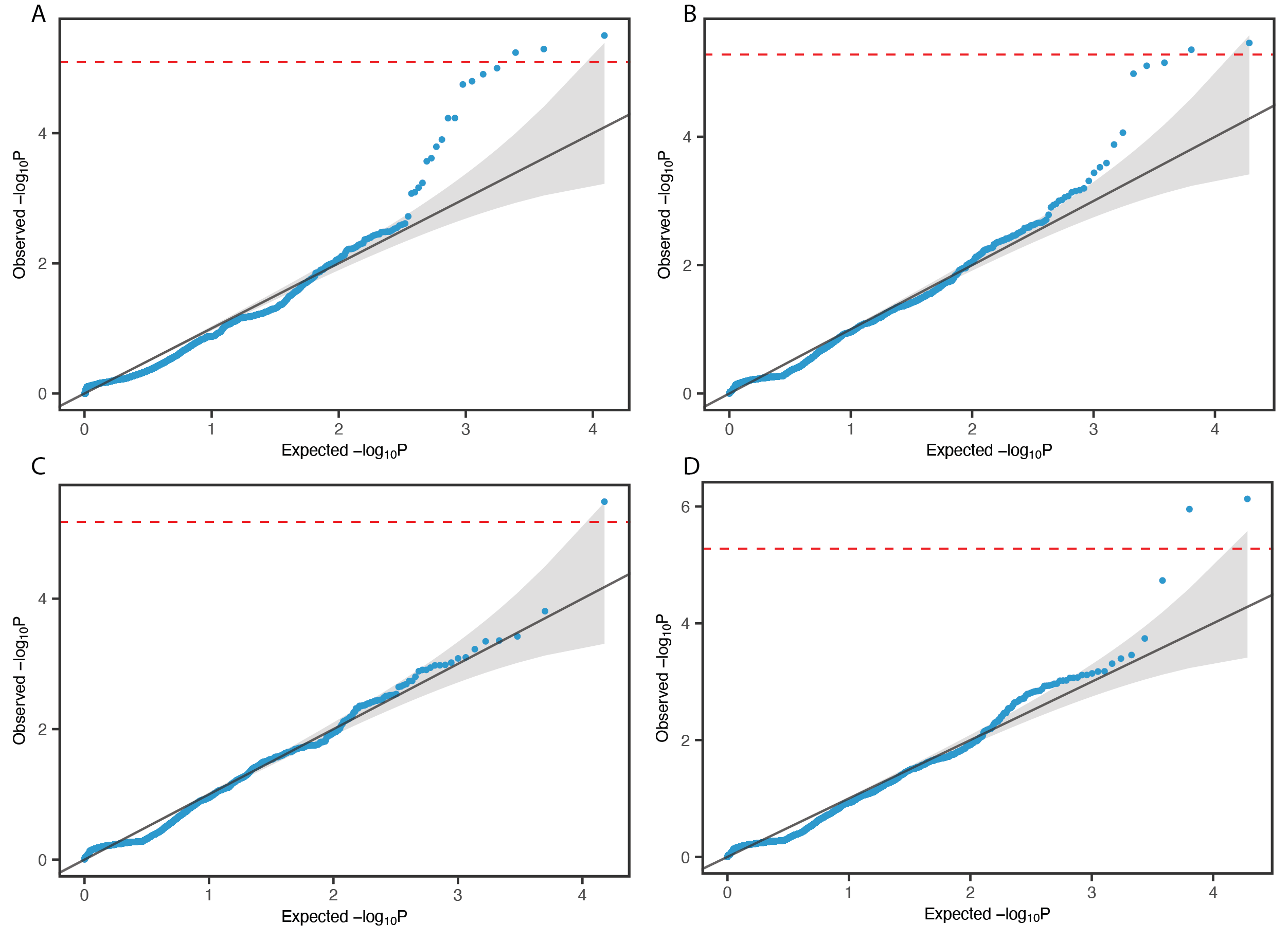


# **Fig. S4.** QQ plots of the SKAT-O results.

**A.** SKAT-O for testing the neutral model. Analysis with 72 cases and 426 controls using synonymous variants resulted in inflated *P* values. **B.** SKAT-O using presumably deleterious variants of the 72 cases and 426 controls, showing inflated *P* values similar to the neutral model. **C**. SKAT-O for testing the neutral model after detecting and excluding 5 samples resulting in inflated *P* values. Inflation seen in the previous analysis was resolved except for one gene, *EPHA4*, which displayed an increased burden on synonymous variants in cases. **D.** SKAT-O using presumably deleterious variants of the 67 cases and 426 controls after the exclusion of the 5 samples.


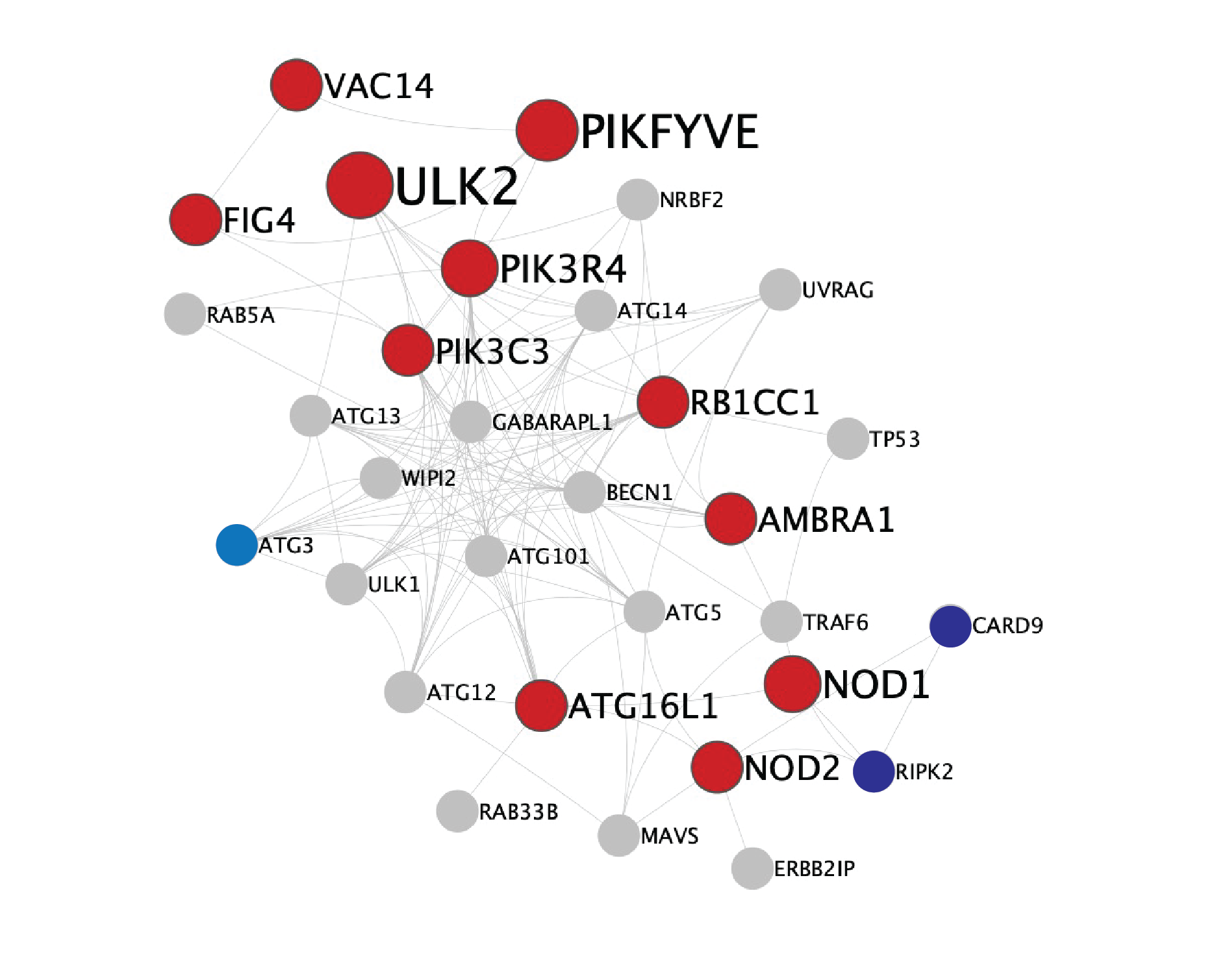


# **Fig. S5.** STRING PPI network of the Cluster73 from NHC analysis.

Genes identified in the Cluster 73 are shown in red. The size of the nodes is proportional to the number of variant carriers. Known IBD- and PD-associated genes in the extended PPI network are highlighted in blue and navy blue, respectively.


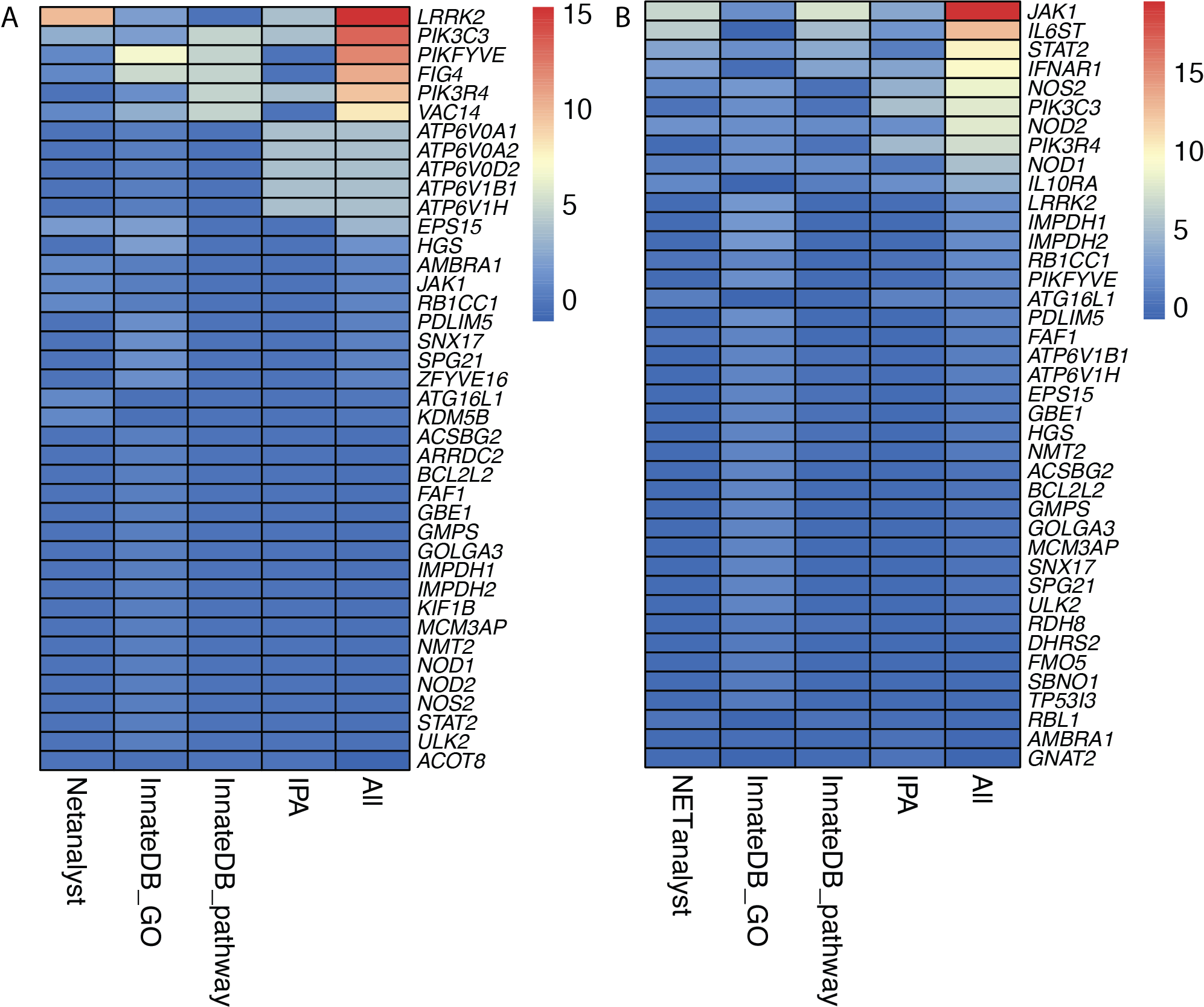


# **Fig. S6.** Heatmap of the combined biological importance scores.

**A.** Scores generated using known PD-associated genes. **B.** Scores generated using known PD-associated genes. A higher score indicates a higher number of shared pathways, ontologies or modules with known PD and IBD genes. The colors represent the magnitude of the scaled scores calculated by each method. The highest scaled scores are depicted in red, whereas the lowest scores are shown in blue. The top 40 genes are shown in the plot.
